# Pioneering Soundscapes: Investigating Commercial Fused Deposition Modelling Filament’s Potential for Ultrasound Technology in Bone Tissue Scaffolds

**DOI:** 10.3390/bioengineering12050529

**Published:** 2025-05-15

**Authors:** Hatice Kübra Bilgili, Masahiro Todoh

**Affiliations:** 1 Division of Human Mechanical Systems and Design, Graduate School of Engineering, Hokkaido University, Sapporo 060-8628, Japan; 2 Division of Mechanical and Aerospace Engineering, Faculty of Engineering, Hokkaido University, Sapporo 060-8628, Japan; todoh@eng.hokudai.ac.jp

**Keywords:** tissue scaffold, ultrasound, bone, fused deposition modeling, poly (lactic acid), polycaprolactone

## Abstract

Daily exposure to various forces creates defects in the musculoskeletal system, leading to health issues, especially for bones. Bone tissue scaffolds and ultrasound technology are both utilized in research and in clinics to enhance bone tissue regeneration. This study aimed to investigate the potential of commercially available fused deposition modeling (FDM) filaments for ultrasound technology using X-ray diffraction (XRD), Raman spectroscopy, nanoindentation, three-point bending, and scanning electron microscopy (SEM) characterization methods. Customized FDM filaments were produced by combining polylactic acid (PLA) FDM filaments with medical-grade polycaprolactone (PCL). Using these, we observed the successful production of complex tissue scaffolds via PLAPCL4060 and PLAPCL5050 FDM filaments. Additionally, the presence of the contrast difference observed via SEM for PLAPCL4060 suggests phase segregation and a material that has both damping and activating characteristics under ultrasound propagation. Mechanical characterization provided hardness and elastic modulus values, while the three-point bending results proved the flexible nature of PLAPCL4060 and PLAPCL5050, which is important for their dynamicity and responsiveness under ultrasound propagation. Accelerated degradation experiments provided crucial information regarding the effect of the porosity and gradients of scaffolds under ultrasound stimulation. Future studies based on this approach will contribute to understanding the true potential of these filaments for bone tissue.

## 1. Introduction

Everyday exposure to different loads is known to affect the human body and cause musculoskeletal diseases [1]. Bones are a prominent component of this system and are often associated with recurrent injuries and illnesses. From both an economic and health perspective, musculoskeletal problems can impose a persistent burden on individual countries. A fracture of the femur can result in job absenteeism, which then triggers a domino effect, i.e., diminished productivity, a reduced quality of life, significant treatment expenses, and sociopsychological issues. These consequences, among others, have prompted researchers to investigate many domains and to propose multidisciplinary solutions for the development of artificial bone structures that are capable of supporting, assisting, and/or replacing bone tissue [2,3,4].

Bone is a type of connective tissue, which is identifiable by its heterogenous composition, both hierarchy- and component-wise; it comprises both organic (e.g., collagen type I) and inorganic compounds (e.g., hydroxyapatite). Bone stores minerals and provides protection and support to other tissues [5,6]. According to the current understanding of bone tissue engineering, bone regeneration requires three main components—cells that can generate a functional matrix; bio-active substances, such as growth factors, which promote specific issues; and scaffolds, which provide mechanical support and cellular adhesion [7].

Consequently, scaffolding is a fundamental element of bone tissue engineering. The objective of bone tissue scaffolds is to emulate the composition and functionality of the natural bone’s extracellular matrix (ECM), facilitating a three-dimensional (3D) environment that encourages adhesion, proliferation, and differentiation, while possessing the requisite physical properties for bone regeneration [8]. Desirable scaffold qualities include biodegradability, biocompatibility, bioactivity, osteoconductivity, and osteoinductivity [4]. Bone scaffolds incorporating biomaterials and additives, including pharmaceuticals [9,10,11], growth factors [11,12,13], and stem cells [14,15,16], have demonstrated efficacy in bone regeneration [17].

Considering the nature of the body, cells that comprise tissues are subjected to a wide range of stimuli, which, in turn, affects their function and fate [18]. Consequently, researchers have been focusing their attention on the development of scaffold designs that have the potential to address the temporal properties of native cells. A great number of studies have demonstrated that dynamic physical stimuli, such as those that are electrical, magnetic, acoustic, and mechanical, have the ability to efficiently control a wide variety of cell responses. On the other hand, extensions to in vivo applications, which require stimuli from the outside, have been restricted [19,20,21,22,23,24]. Ultrasonic stimulation is one of the most frequently utilized methods in clinical settings for the sake of both therapeutic [25] and diagnostic applications [26], having been used for a number of years [24]. From a cell behavior perspective, as seen in clinics and the literature, ultrasound stimulation has been used as a tool for supporting and advancing the healing process of defected tissue. In particular, one research paper that focuses on the cell behavior under ultrasound effect can explain the potential outcomes. As pointed out by the referenced article, cells under ultrasound stimulation can experience translation, proliferation, apoptosis, lysis, oscillation, and transient membrane permeation [27]. Additionally, they have been reported to observe internal and cytoskeletal changes in cells. In light of the previously mentioned clinical uses, ultrasound has been used as a tool for bone tissue healing (in the form of low-intensity pulsed ultrasound) and as a source of mechanical stimulation that can activate the integrin/phosphatidylinositol 3-OH kinase/Akt pathway and upregulate osteogenic proteins by producing prostaglandin E and cyclooxygenase-2 (COX-2) [28]. Thus, in the presence of ultrasound stimulation, such behaviors from the cell have the potential to be observed.

Researchers have recently begun investigating the possibility of using ultrasound stimulation (US) in conjunction with tissue scaffolds to enhance tissue growth and regeneration. The majority of these studies have focused on the interaction between ultrasound waves and developing tissue, while ignoring the key factor of mechanical stimulation, which results from the waves interacting with the scaffold [24,29,30]. Additionally, a great number of studies have utilized finite element analysis (FEA) to investigate the various effects of ultrasound (such as in the form a tool for investigating material–ultrasound wave interactions and tissue scaffold–ultrasound wave interactions). Loving et al. revealed that the fiber orientation and defect size in bioinspired composites substantially influenced the energy and propagation direction of ultrasonic waves within the composite [31], while Zhao et al. employed Mia scattering as an analytical tool to evaluate the validity of both experimental and computational research regarding ultrasonic wave propagation in tissue scaffolds [32]. Thus, it was observed that scattering predominantly occurs at the scaffold surface and that reduced pore size and lower wave signal frequencies facilitate the transmission of wave energy through the scaffold. A subsequent study examined the peak density, which is defined as the number of local peaks and troughs in the frequency power spectrum of the ultrasonic signal following its transmission through a material [33]. Furthermore, researchers have also utilized ultrasound technology for PLA and PCL scaffolds as an element for stimulation as part of the production process. Khodaei et al. utilized ultrasound vibrations as part of the process of 3D printing in order to inhibit any clogging that occurs due to the presence of nanoparticles. For this research, PLA scaffolds containing different percentages of akerminite were printed via the Fused Filament Fabrication method. The authors reported that ultrasonic-assisted 3D printing is an improved manufacturing process that can overcome the potential limitations resulting from viscosity [34]. Another approach that used ultrasound as part of its production technique was investigated by Olmo et al., who utilized PLA, PLA:PEG, and PLA with NaCl nanoparticles and ultrasound as part of a molding process in the production of scaffolds [35]. In summary, the current literature focuses on different materials and techniques, with the purpose of using ultrasound in combination with tissue scaffolds. Nevertheless, one study focuses on bone regeneration via the use of two commonly used biopolymers—polycaprolactone (PCL) and polylactic acid (PLA). Camarero-Espinosa et al. manufactured PLA/PCL blends in different percentages in order to achieve phase segregation, which was the main application for ultrasound technology. They further discovered that in the phase-segregated PCL/PLA blend (50:50), one polymer acts as a damping element (PCL), while the other one acts as a deflecting element (PLA). They named these types of scaffolds Janus scaffolds (named after Janus particles), and experimental results showed that under US simulation, increases in cell proliferation, osteogenic differentiation, and the matrix deposition of bone marrow-derived stromal cells (BMSCs) were achieved [24]. However, there are a few issues that need to be further investigated. First, the tissue scaffolds that were used during experiments were scaffolds with a uniform porosity and a single layer. As has been established, the biomimicry of bone tissues is complicated due to the nature of bone, and a significant amount of research has been conducted to try and achieve optimal properties (pore size, porosity percentage, geometrical structure, etc.). Another problem that needs to be pointed out is the manufacturing method. The 3D printer that was used for this study deposits pellets by melting them directly onto the surface, and both polymers were purchased in pellet forms. By adapting this manufacturing method into filament-based FDM, 3D printers would improve the availability and accessibility of such a technology. Such potential improvements have led us to conduct the current study. This study aims to address the following research gaps: the limitation of the current literature in relation to ultrasound-responsive scaffold manufacturing methods lacking accessibility; the need for the adaptation and application of ultrasound technology (e.g., low ultrasonic vibrations sourced from an ultrasound generator) with FDM filaments and FDM printing for improved accessibility; the need for research regarding ultrasound technology applications with tissue scaffolds that possess a complex geometry (e.g., TPMS and functionally gradient), i.e., the need to investigate and understand scaffold and ultrasound wave interactions for these scaffolds and what these interactions can mean for bone tissue scaffolds; and, due to the current literature lacking focus on scaffold geometry complexity, the need for further investigation into how ultrasound interacts with such complexity in scaffold layers as well as the geometry of the scaffolds.

In this study, the potential of commercial FDM filaments for ultrasound technology in bone tissue scaffolds was investigated within the scope of the key elements of scaffold composition (i.e., the production of ultrasound-responsive materials), mechanical characteristics (e.g., flexural modulus, hardness, and elastic modulus), and printability (i.e., the potential to be adapted into additive manufacturing). These key elements are based on both the current ultrasound-based studies and the essential characteristics of bone tissue scaffolds. With this aim, by using commercialized PLA FDM filaments and medical-grade PCL, the following aspects were investigated: (1) the adaptability of the suggested technology to FDM printing via the production and analysis of printing conditions; (2) the presence of phase segregation between PCL and PLA polymers, which creates the dynamic nature of a scaffold, via X-ray diffraction (XRD), Raman spectroscopy, and scanning electron microscopy (SEM); (3) the mechanical characteristics of polymer blends compared to commercial PCL and PLA filaments via nanoindentation testing; (4) the flexural modulus of PLAPCL4060 and PLAPCL5050 to determine responsiveness under ultrasound propagation; and (5) the potential of the proposed technology in bone tissue scaffolds via investigating the accelerated degradation of TPMS scaffolds with uniform, x/y, and radial gradients in the presence of ultrasound stimulation.

## 2. Materials and Methods

### 2.1. Production of Customized FDM Filaments

**Figure 1:**
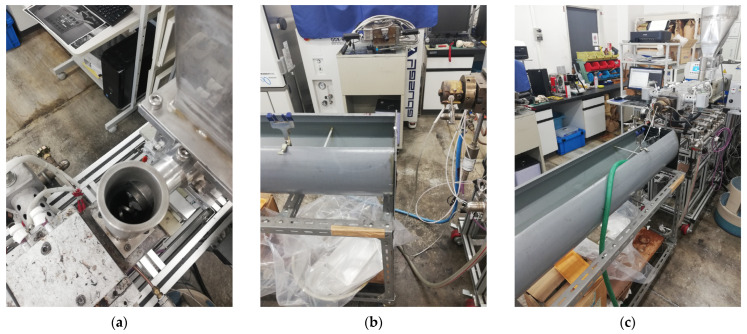

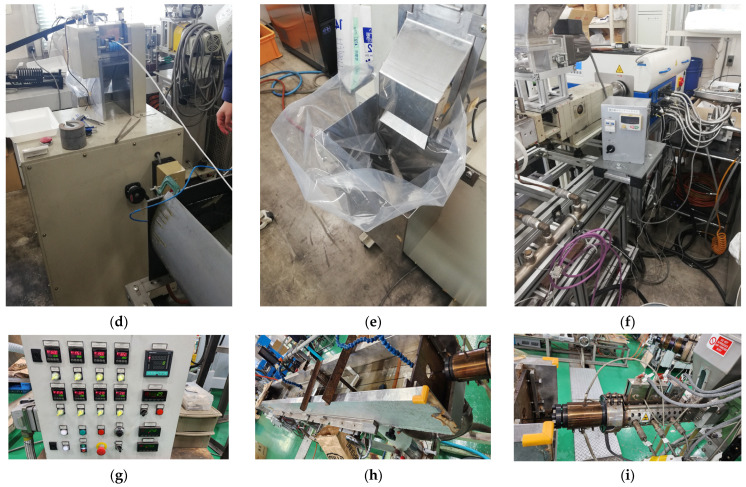
Production of PLA pellets and PLAPCL mixed pellets for the second screw extrusion (**a**–**f**). The second extrusion of FDM filaments of PLAPCL4060 and PLAPCL5050 for uniform diameter and polymer blending. (**a**) Locations where the polymers were fed into the machine; (**b**) the beginning of extrusion point; (**c**) the path of extruded filament and the cooling of the filament; (**d**) the final part of the extrusion and the pelleting head; (**e**) the pelleted mixed PLAPCL filament; (**f**) the control panel part of the extrusion machine, (**f**) The second extrusion conditions and the extrusion of the filaments, (**g**) the control panel of second extruder, (**h**) the pathway for extruded filament during production, (**i**) the feeding part of the extruder.

PCL (MW: 80,000 Da; 1.145 g/mL at 25 °C; Sigma-Aldrich, St. Louis, MO, USA and Raise3D Premium PLA Filament (white) (density: 1.2 g/cm^3^ at 21 °C; Raise3D Nantong, China) were purchased. As shown in Figure 1, the PLA was cut into small flakes. The two polymers were weighed and mixed, before being fed into an extruder at 150 °C at 100 rpm [3] to produce the necessary PLA: PCL ratios of 40:60 wt./wt. and 50:50 wt./wt. A second extruder was used to achieve a 1.75 mm standard FDM filament diameter. The mixtures were further shredded into tiny pieces and put into another extruder, with conditions being based on the reference conditions of 150 °C at 100 rpm; two separate conditions were tested. Firstly, the following conditions were tested: cylinder at C1: 160 °C, C2: 175 °C, and C3: 190 °C; adapter at 185 °C and die at 170 °C; screw speed at 24.8 rpm; extruder diameter of 12 mm; and extruder speed at approximately 3.7 mm. Secondly, the following conditions were tested: cylinder at C1: 150 °C, C2: 165 °C, and C3: 175 °C; adapter at 170 °C and die at 165 °C; screw speed at 20.0 rpm; extruder diameter at 12 mm; and extruder speed at approximately 3.5 m/min. With the second set of conditions, a uniform fiber diameter was achieved. The repetition of extrusion steps with different extruders was conducted to achieve a uniform filament diameter.

### 2.2. Analysis of the Printing Conditions for FDM Filaments

**Figure 2:**
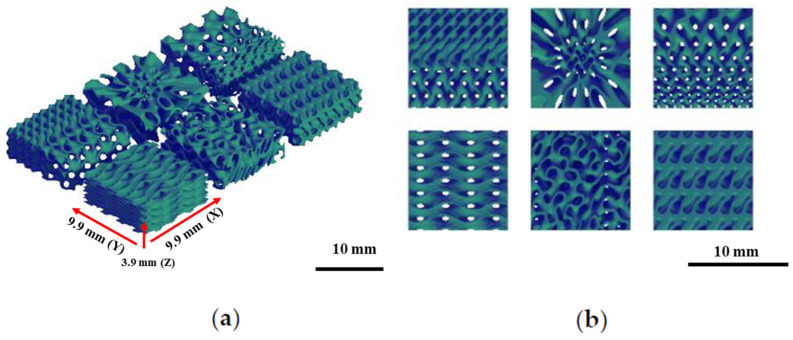
STL images of example scaffolds used for printing analysis [37]. (**a**) Perspective view; (**b**) top view.

The printing conditions for mixed PLA/PCL custom filaments were tested according to the PLA printing temperature of 205 °C. A lower temperature (150 °C) and high temperatures (180 °C or 200 °C) were chosen and a Raise3D Pro2 FDM printer (Raise 3D, Irvine, CA, USA) was used. The samples printed at these different temperatures were analyzed via scanning electron microscopy. Additionally, to test the resolution capability of the FDM printer, tissue scaffolds with varying complexities were printed (Figure 2) using PLAPCL4060, PLAPCL5050, and PLA filaments (Raise3D Premium PLA filament, blue). Tissue scaffolds were outsourced from the literature [36]. Example Grasshopper files that were provided on the Axolotl plug-in were used directly [37], while STL files were exported via Grasshopper into Rhinoceros^®^ (Robert McNeel & Associates, Seattle, WA, USA).

### 2.3. Customized Filament Characterization

#### 2.3.1. X-Ray Diffraction

The crystallization and microstructure of PLAPCL4060, PLAPCL5050, PLA (Rasis3D Premium PLA filament, blue), and PCL (RoHS PCL filament, sky blue) were analyzed via Rigaku-Smartlab (Rigaku, Tokyo, Japan) XRD using CuKα radiation with a wavelength of 1.542; a beam configuration set-up of 4 and 50°, which continues to 2θ values; a 0.020° resolution; and a scanning speed of 2.00°/mm. For all four main groups, both the printed and filament versions were analyzed using XRD. The filament samples were 1.75 mm thick, while the printed samples were 2 mm thick.

#### 2.3.2. Raman Spectroscopy

Raman spectroscopy was conducted on the filament (1.75 mm thickness) and printed samples (2 mm thickness) of PLAPCL4060, PLAPCL5050, PLA (Rasis3D Premium PLA filament, blue), and PCL (RoHS PCL filament, sky blue) using inVia Reflex (Renishaw, UK) with an excitation laser wavelength of 532 nm. The experimental parameters included 1100 Raman shift/cm^−1^, 10% power, and a wavenumber range of 50–1800 cm^−1^. The scanned results were analyzed via Origin2024 b (Origin Lab Corporation, Northampton, MA, USA), with the PLA and PCL databases. For smoothing the data, the Savitzky–Golay Filter was used with a polynomial order of 2 and a window size of 5 points. Additionally, an open-source spectrometer analysis software program (Open Specy) was used; preprocessing (threshold signal–noise: min value at 4; min–max normalization; smoothing/derivative; conform wave numbers) and identification (ID library: AI:Both Deriv Mediod) were executed [38].

#### 2.3.3. Morphological Analysis with Scanning Electron Microscopy

FE-SEM with EDS (JIB-4601F, JEOL, Tokyo, Japan) at 5 kV and 8.00 mm WD was employed to characterize the surface microstructures of the PLAPCL4060, PLAPCL5050, PLA, and PCL FDM filaments and their printed versions (grid and TPMS). The samples were precisely cut into dimensions according to the sample holder, followed by a coating process using a JFC-1600 Auto Fine Coater for a duration of 180 s at a current of 20–30 mA.

#### 2.3.4. Mechanical Characterization with Nanoindentation

A nanoindentation analysis was performed using an Elionix ENT-1100a (Elionix Inc. Tokyo, Japan) Nano indenter with a Berkovich-type tip, with diameters of 2 mm × 10 mm × 10 mm, for PLAPCL4060, PLAPCL5050, and PLA. The indenter was placed at a 900–1000 μm distance, and the indentation depth was set to 900–1000 μm with a maximum load of 150 μN. An average of the points was tested (3 samples × 10 points). Statistical analyses were conducted using one-way ANOVA in Origin2024b (OriginLab Corporation, Northampton, MA, USA). Additionally, standard deviations were calculated in Excel using the stdev. p function.

#### 2.3.5. Three-Point Bending Test Using a Universal Testing Machine (UTM)

The three-point bending test was conducted using an Instron 3365 UTM (Instron, Norwood, MA, USA) equipped with a 50 N load cell and controlled with the Bluehill software 2.17 program in order to calculate the flexural moduli of PLAPCL4060, PLAPCL5050, and PLA. Because the FDM filament dimensions were unsuitable for testing, structures with dimensions of 2 mm × 10 mm × 30 mm were 3D-printed for testing. Tests were repeated for three samples from each polymer mixture, and the flexural modulus was calculated using the average slope from the load–displacement curves. Statistical analysis was conducted using one-way ANOVA in Origin2024b (OriginLab Corporation, Northampton, MA, USA). Additionally, standard deviations were calculated using the stdev. p function in Excel.

### 2.4. Application of Ultrasound for Bone Tissue Scaffolds

#### 2.4.1. Design and 3D Printing of Triply Periodic Minimal Surface Scaffolds

**Figure 3:**
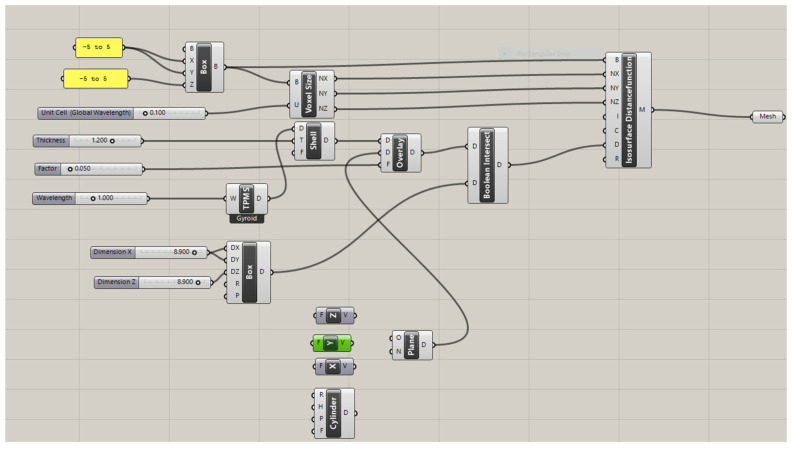
Workflow of uniform, radial, and x/y gradient TPMS gyroid scaffolds in Grasshopper.

Triply Periodic Minimal Surface (TPMS) scaffolds were designed using Rhino and Grasshopper with the Axolotl plug-in. The design was based on defining the TPMS as the input lattice and the principal stress concentration as the modifying field [36]. As a reference, example files were used, and the final modified workflow, which is given in Figure 3, was constructed [36,37]. Based on the parameters of a global wavelength of 0.100, a thickness of 1.200, a wavelength of 1,00 for the gyroid function, and dimensions of (x, y, z) as (8.9 mm, 8.9 mm, 8.9 mm), the TPMS structures were design with gyroid unit cells with a gradients of uniform, x/y, and radial gradients (Figure 3). For the x/y gradients, an x/y vector was added as input; for the radial gradients, a cylinder element was added. The workflow given in Figure 3 can be briefly explained as follows. First, a regular periodic microstructure was assigned as the signed distance function—in this case, it is the TPMS gyroid. Then, modifiers are introduced to change the parameters of the base lattice. For this workflow, the modifiers used are global wavelength, thickness overlay, shelling, and coordinate system. By multiplying x, y, and z by the appropriate factor, one can alter the global wavelength—the distance after which the pattern periodically repeats—or the size of the unit cell. Either a uniform value or a non-uniform value with distinct values for each axis can be used for this, since the minimum surface of a TPMS separates a volume equally into void and solid. Shelling gives the surface a solid thickness by dividing a volume into two interwoven but unconnected voids. It also includes t (thickness, which increases the SDF’s distance) and D (distance, which is the TPMS). The box is the part that assigns the dimensions of the TPMS and overlay (thickness overlay). By adding a value to the output of the TPMS equation, the geometry can be eroded (positive value) or dilated (negative value), which alters the location of the 0-level isocontour. As is the case for the wavelength, this can be performed locally or globally using a field. As shown in Figure 3, this overlay also controls the distribution of pore structures, which can result in uniform, radial, and x/y gradients, among others. Boolean intersection creates a variety of boundary conditions along the contour by cutting through the lattice pattern at wildly varied angles. Using the marching cubes method, the Isosurface Distance Function meshes the object before using the right-click and bake option to bake it into Rhino [36].

**Figure 4:**
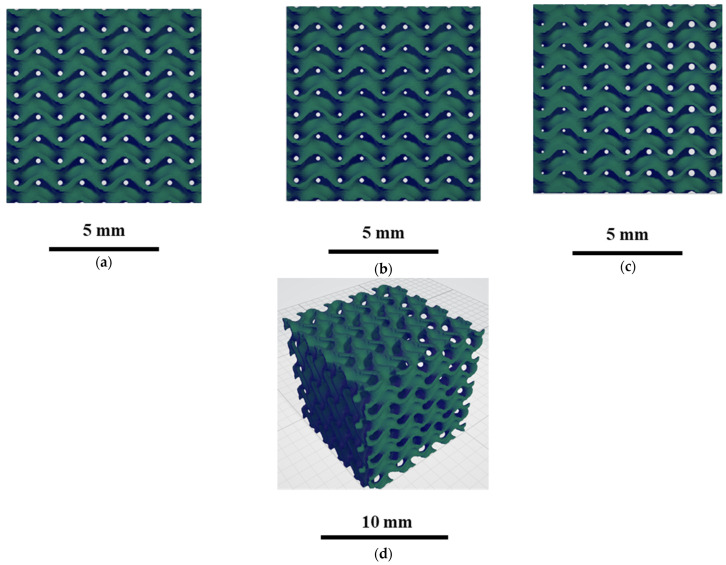
CAD design of TPMS gyroid scaffolds. Top view of (**a**) scaffold 1 (S1) with uniform gradients; (**b**) scaffold 2 (S2) with radial gradients; (**c**) scaffold 3 (S3) with x/y gradients; and (**d**) isometric projection of 3D TPMS scaffolds.

The scaffolds designed based on this workflow are given in Figure 4 for (a) uniform, (b) radial, and (c) x/y gradient scaffolds.

TPMS scaffolds were printed with a Raise3D Pro2 FDM printer (Raise 3D, USA) using PLAPCL4060, PLAPCL5050, and PLA filaments. The printing conditions given in Table 1 were optimized for TPMS structures for each filament type. For PLA, the parameters suggested by the supplier were used (see Supplementary Materials for more details).

#### 2.4.2. Accelerated Degradation of Scaffolds Under Ultrasound Stimulation

**Figure 5:**
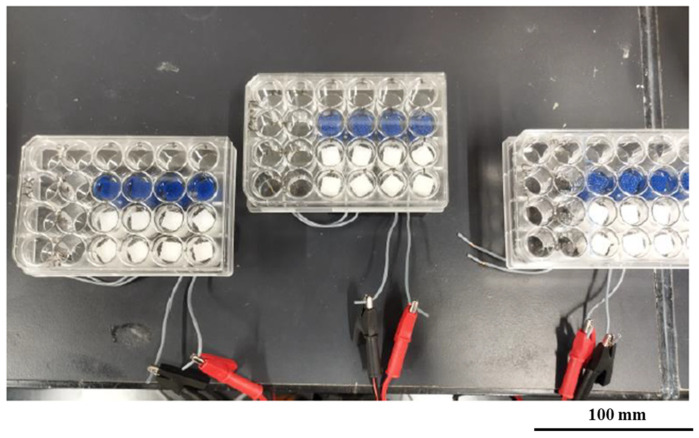
Ultrasound was applied to scaffolds by placing an ultrasound source under 24-well plates.

Accelerated degradation experiments were conducted. TPMS scaffolds with uniform (S1), radial (S2), and x/y (S3) gradients were immersed in 5M NaOH (Sigma-Aldrich) [39] in 24-well plates and were incubated at 37 °C for a total of 6 hours (h) for both ultrasound-applied (US) and non-ultrasound-applied (NUS) samples of 4060PLAPCL, 5050PLAPCL, and PLA. Ultrasound stimulation was applied twice at 40 kHz with a Kemo 8071 for 1 h during the experiment at room temperature (for US samples: 2 h ultrasound stimulation at room temperature (RT) and a total of 6 h incubation at 37 °C) with the set-up shown in Figure 5. At 1, 3, and 6 h, the weights of each sample were measured. Additionally, the initial and final dry weights of the samples (dried at 37 °C for 24 h and at room temperature for 48 h) were measured using an electronic balance; then, the mass loss percentage was calculated.

#### 2.4.3. Morphological Analysis of Degraded Samples Using Scanning Electron Microscopy

FE-SEM with EDS (JIB-4601F, JEOL, Tokyo, Japan) at 5 kV and 8.00 mm WD was employed to characterize the surface microstructures of US and NUS PLAPCL4060, PLAPCL5050, and PLA samples (S1, S2, and S3). The samples were coated using a JFC-1600 Auto Fine Coater for 180 s at 20 mA. The surface structures after degradation, as well as the degradation pattern differences between the NUS and US samples, were observed and reported.

## 3. Results

### 3.1. Production of Customized FDM Filaments

**Figure 6:**
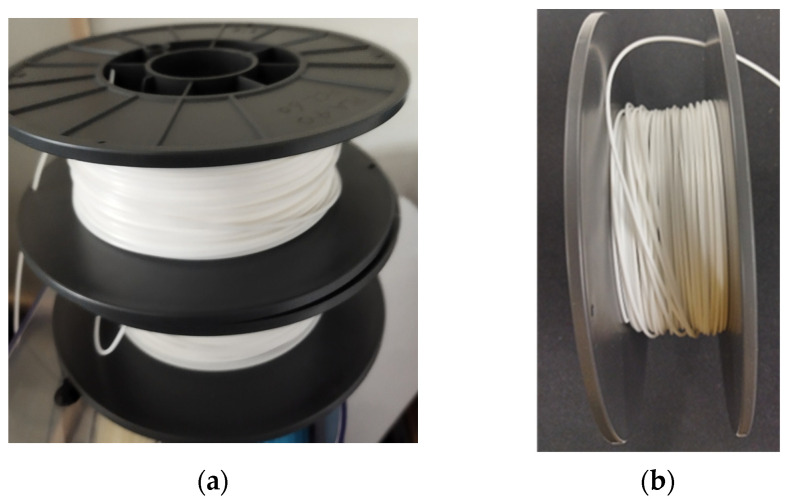
Final products of PCL/PLA customized filaments. (**a**) The view from the front of PLAPCL4060 and PLAPCL5050; (**b**) cross-sectional view of customized filaments.

The final products of the PLAPCL4060 and PLAPCL5050 samples are shown in Figure 6. Each filament has a diameter of 1.75 mm and weighs approximately 800–1000 g.

### 3.2. Analysis of Printing Conditions of Customized FDM Filaments

**Figure 7:**
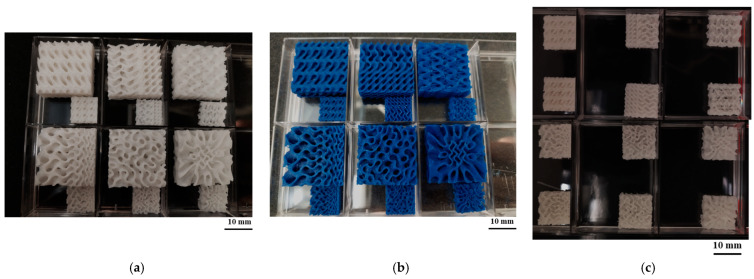
Bone tissue scaffolds with varying complexities using (**a**) PLAPCL5050 (200%); (**b**) PLA (200%); and (**c**) PLAPCL 5050 vs. PLAPCL4060 printed at a 125% scale.

The impact of printing conditions on the PLAPCL4060 and PLAPCL5050 filaments was tested using geometrical complexity and porosity variance. To investigate the limitations of 3D printing, which could be related to the FDM printer and/or the FDM filament itself, scaffolds were printed at different scales, as shown in Figure 7a,b. In Figure 7c, scaffolds of the same size were printed using both PLAPCL4060 and PLAPCL5050 to investigate the abovementioned limitations. The structures shown in Figure 7 reveal that as the complexity increases, the precision and quality within the overall structure are difficult to maintain using 3D printing for the FDM filaments of PLAPCL4060 and PLAPCL5050 when compared to the filaments of PLA (the observed defects and alignments reported can be found in the Supplementary Materials). Additionally, as seen in Figure 7a,c, when compared, the two PLAPCL mixture-based structures also have differences in precision and quality.

### 3.3. Customized Filament Characterization

#### 3.3.1. X-Ray Diffraction

**Figure 8:**
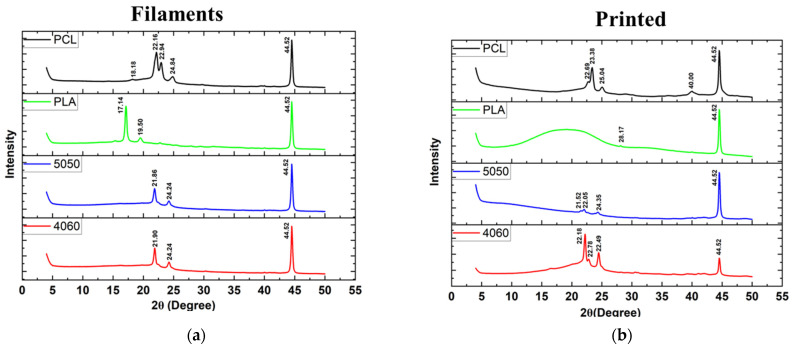
XRD peaks for PLAPCL4060, PLAPCL5050, PLA, and PCL either as (**a**) filaments or (**b**) printed.

Figure 8 shows the peaks detected via XRD spectroscopy. The peaks of the filaments were as follows: for PLAPCL4060, the peaks were 22.16°, 22.78°, and 44.52°; for PLAPCL5050, they were 22.05°, 24.35°, and 44.52°; for PLA, the peak was 44.52°; and for PCL, the peaks were 23.26°, 25.04°, 40.60°, and 44.52°. For the printed structures, the detected peaks were as follows: for PLAPCL4060, they were 21.90°, 22.75°, and 44.52°; for PLAPCL5050, they were 21.86°, 24.24°, and 44.52°; for PLA, they were 17.14°, 19.50°, and 44.52°; and for PCL, they were 22.16°, 22.94°, 24.90°, and 44.52°. Further outcomes based on the literature for PLA and PCL peaks have been further discussed in the Discussion section.

#### 3.3.2. Raman Spectroscopy

**Figure 9:**
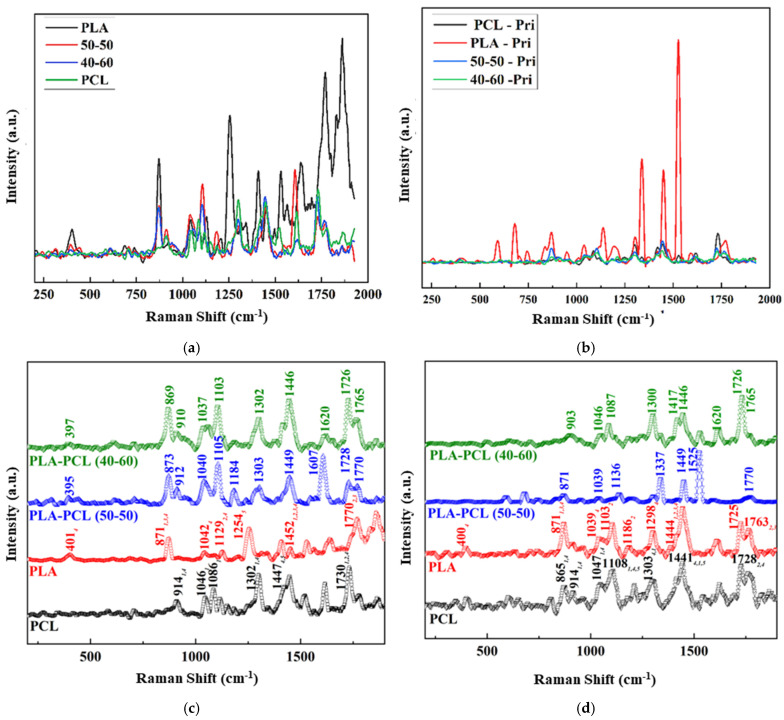
(**a**) Raman spectra of filaments of PLA, PLAPCL4060, PLAPCL5050, and PCL (smoothed and plotted); (**b**) printed spectra of PLA, PLAPCL4060, PLAPCL5050, and PCL (smoothed and plotted); (**c**) filament spectra of PLA, PLAPCL4060, PLAPCL5050, and PCL (normalized); and (**d**) printed spectra of PLA, PLAPCL4060, PLAPCL5050, and PCL (smoothed).

Raman spectrum analysis derived from both Origin and databases detected peaks corresponding to PLA and PCL, as shown in Figure 9. However, the following peaks showed shifts from the references of 0–6 cm^−1^ [40,41,42,43,44,45,46,47,48]. For filaments, the matches detected were as follows: PLAPCL4060 matched with PLA peaks of 397 cm^−1^, 869 cm^−1^, 1037 cm^−1^, 1103 cm^−1^, and 1765 cm^−1^, as well as with PCL peaks of 91 cm^−1^, 1302 cm^−1^, 1446 cm^−1^, and 1726 cm^−1^; PLAPCL5050 matched with PLA peaks of 395 cm^−1^, 873 cm^−1^, 1040 cm^−1^, 1105 cm^−1^, and 1770 cm^−1^. PLA and PCL FDM filaments matched with the referenced database peaks as follows: PLA matched with 401 cm^−1^, 871 cm^−1^, 1042 cm^−1^, 1129 cm^−1^, 1254 cm^−1^, 1452 cm^−1^, and 1770 cm^−1^; PCL matched with 914 cm^−1^, 1046 cm^−1^, 1302 cm^−1^, 1447 cm^−1^, and 1730 cm^−1^. For printed filaments, the matches detected were as follows: PCLPLA4060 matched with Raman shift peaks of PLA at 1046 cm^−1^, 1087 cm^−1^, 1446 cm^−1^, and 1765 cm^−1^, as well as the PCL peak at 1726 cm^−1^. PLAPCL5050 matched with Raman shift peaks of 1303 cm^−1^, 1449cm^−1^, and 1728cm^−1^.

#### 3.3.3. Morphological Analysis with Scanning Electron Microscopy (SEM)

**Figure 10:**
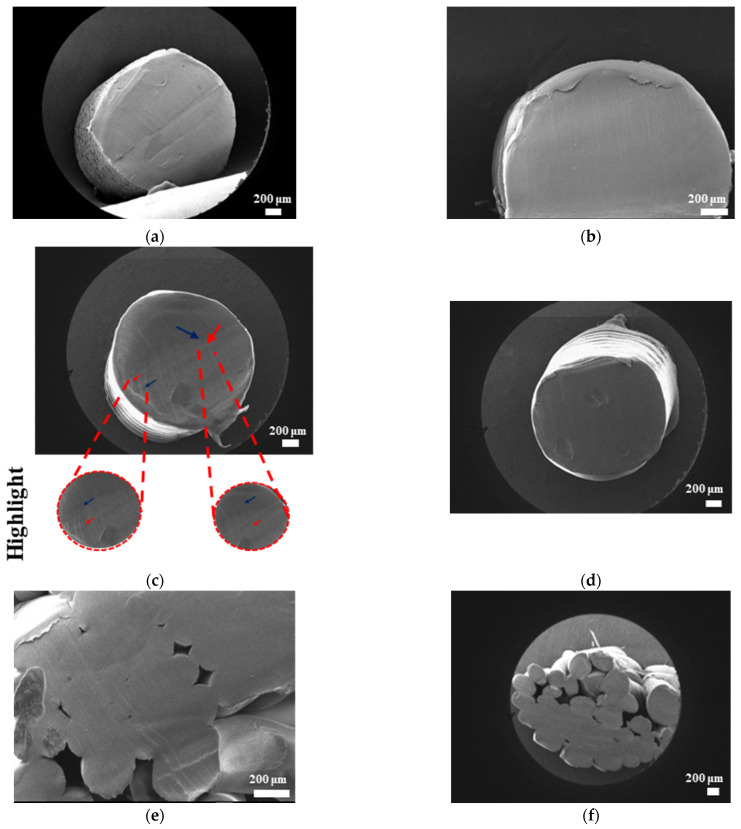

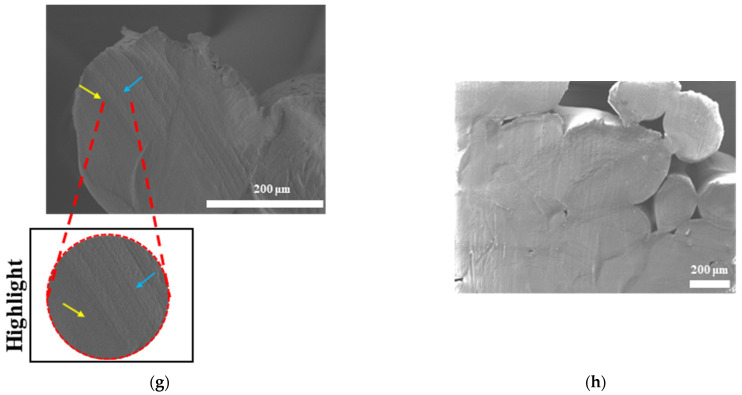
SEM images of filaments before printing: (**a**) PLA, (**b**) PCL, (**c**) PLAPCL4060, and (**d**) PLAPCL5050. SEM images of filaments when printed: (**e**) PLAPCL4060 at low temperature (150 °C), (**f**) PLAPCL5050 at low temperature (150 °C), (**g**) PLAPCL4060 at high temperature (200 °C), and (**h**) PLAPCL5050 at high temperature (180 °C). (Red arrow: bright parts; black arrow: blue parts (phase segregation); yellow and light blue arrows: areas with structural differences.).

**Figure 11:**
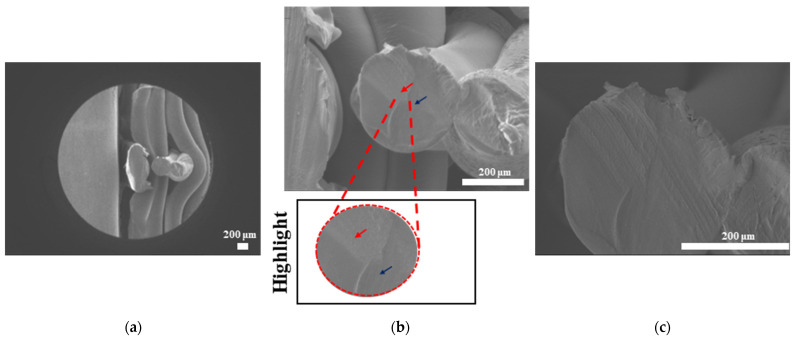

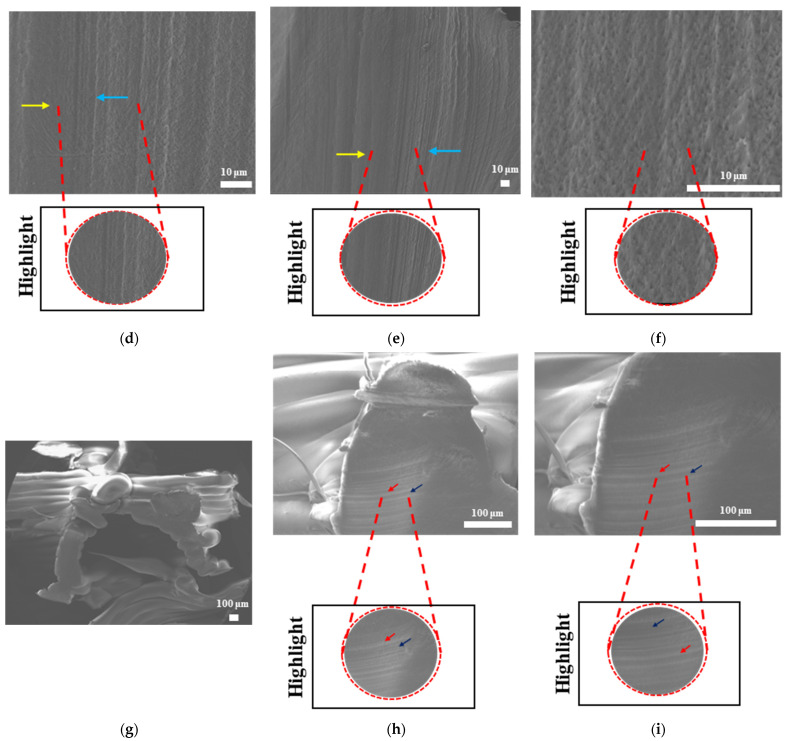
Scanning electron microscopy images of PLAPCL4060 FDM filament structures (**a**–**i**). All structures were printed at 200 °C. (Red arrow: bright parts; black arrow: blue parts (phase segregation); yellow and light blue arrows: areas with structural differences.).

**Figure 12:**
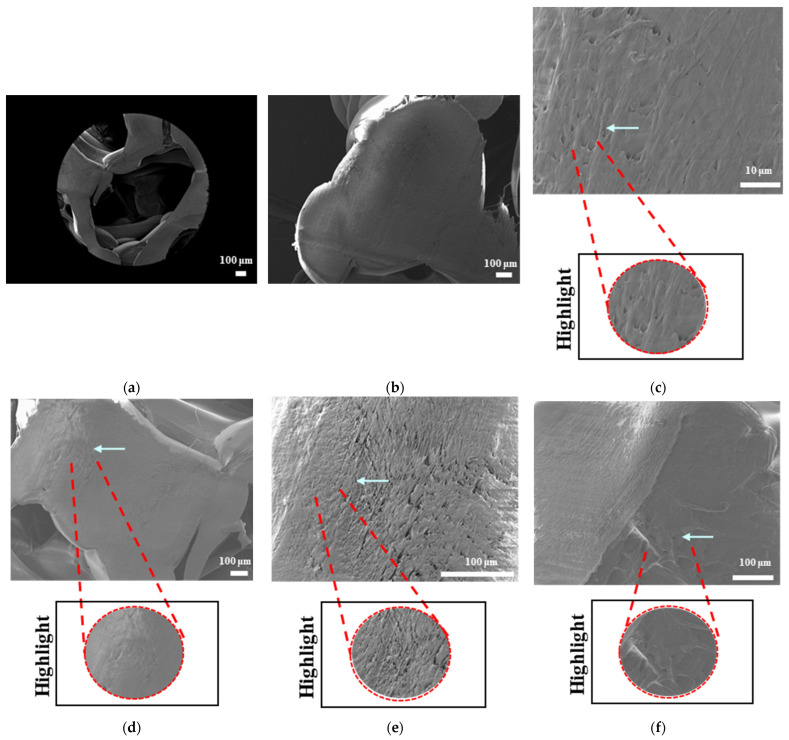

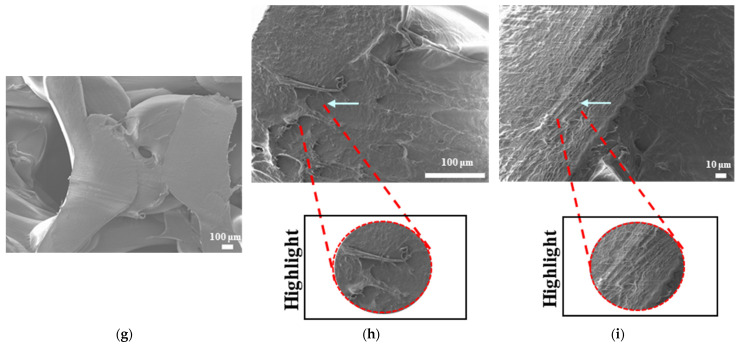
Scanning electron microscopy images of PLAPCL5050 FDM filament structures (**a**–**i**). All structures were printed at 200 °C. (light blue arrows: areas with structural differences.).

**Figure 13:**
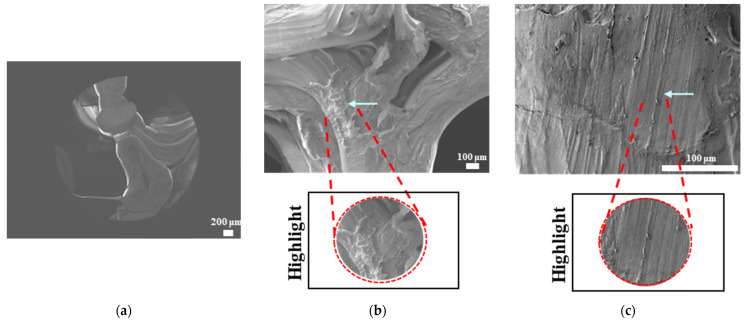

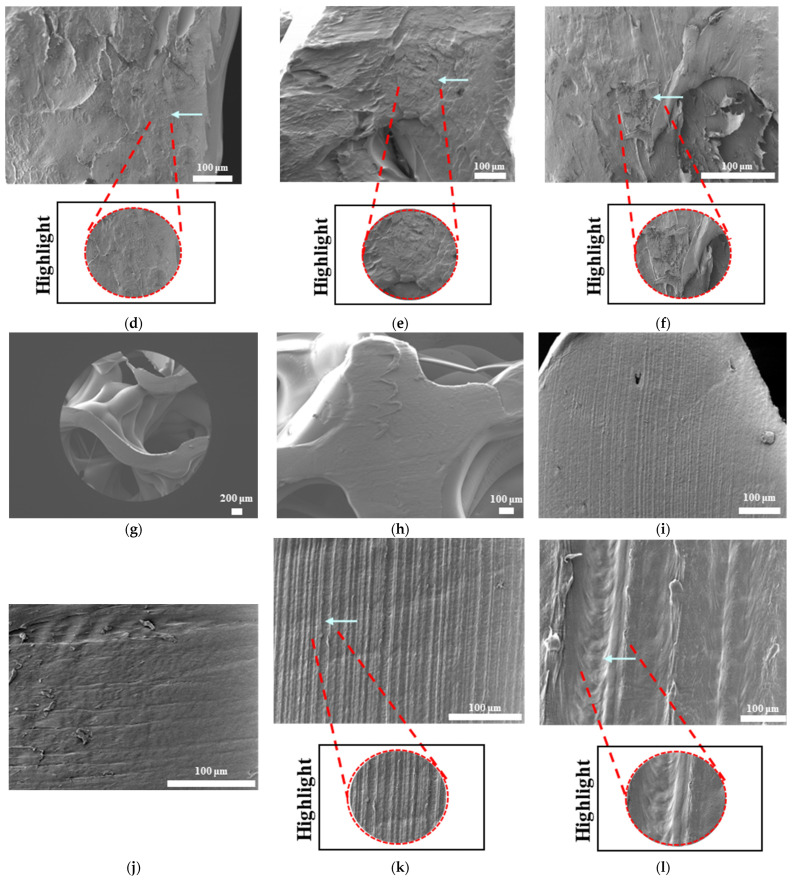
Scanning electron microscopy images of printed PLA (**a**–**f**) and PCL (**g**–**l**). (light blue arrows: areas with structural differences.).

The SEM results for filaments printed at high and low temperatures and printed samples of PLAPCL4060, PLAPCL5050, PLA, and PCL structures are shown in Figure 10, Figure 11, Figure 12, and Figure 13, respectively. The filaments of PLA, PCL, and PLAPCL5050 have homogenous surface structures (Figure 10a,b,d), while a contrast difference was observed for PLAPCL4060 (Figure 10c). For structures printed at low (150 °C) and high temperatures (180–200 °C) with PLAPCL4060 and PLAPCL5050 FDM filaments, a surface structure difference was observed for PLAPCL4060 (Figure 10g) and no significant difference was observed for PLAPCL5050 structures (Figure 10f,h).

For structures that were 3D-printed with PLAPCL4060 and PLAPCL5050 FDM filaments, the following results were obtained. The SEM analysis of PLAPCL4060-based structures revealed a nonhomogeneous surface structure (Figure 11d–f). Additionally, in Figure 11b,h,i,l, contrast differences between the regions of sample surfaces were observed. For PLAPCL5050, a variance of different structures was observed, as shown in Figure 12c–d,h,i. However, no contrast difference was observed.

For PLA printed structures (Figure 13b–f), a nonhomogeneous surface structure was observed throughout the sample, while for PCL printed structures (Figure 13i–l), dents and peaks lying parallel to each other and creating a nonhomogeneous surface structure were observed.

#### 3.3.4. Mechanical Characterization with Nanoindentation

**Figure 14:**
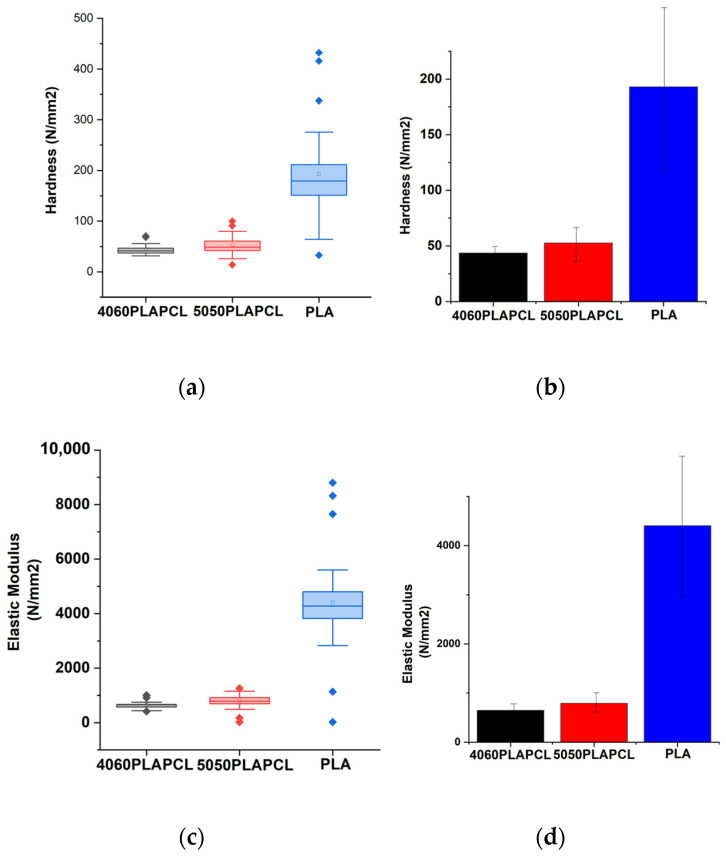
Results of the statistical analysis of the nanoindentation test for printed FDM samples. (**a**) The hardness range for PLAPCL4060 (4060PLAPCL), PLAPCL5050 (5050PLAPCL), and PLA. (**b**) Bar chart of the hardness of PLAPCL4060 (4060PLAPCL), PLAPCL5050 (5050PLAPCL), and PLA. (**c**) The elastic modulus range for PLAPCL4060 (4060PLAPCL), PLAPCL5050 (5050PLAPC), and PLA. (**d**) Bar chart of the elastic modulus of PLAPCL4060 (4060PLAPCL), PLAPCL5050 (5050PLAPCL), and PLA.

The results of mechanical characterization via nanoindentation testing are shown in Figure 14, Table 2 and Table 3. Data points (PLAPCL4060: 29 points; PLAPCL: 67 points; and PLA: 58 points) were used for statistical analysis, and averages were calculated for the hardness and elastic modulus of each. Additionally, due to the limitations of 3D printing with PCL filaments, mechanical characterization with nanoindentation did not include PCL samples. The hardness values for printed structures were as follows: PLAPCL4060—43.62 ± 9.24 MPa; PLAPCL5050—52.58 ± 15.69 MPa; and PLA—92.98 ± 74.99 MPa. The elastic modulus values found were as follows: PLAPCL4060—48.30 ± 117.66 MPa; PLAPCL5050—792.00 ± 214.65 MPa; and PLA—4406.49 ± 1449.7 MPa. Statistical differences between the hardness and elastic modulus values were calculated using one-way ANOVA (Table 2 and Table 3). A statistically significant difference was found between PLA-PLAPCL4060 and PLA-PLAPCL50505. However, there was no significant difference found between PLAPCL5050 and PLAPCL4060.

#### 3.3.5. Three-Point Bending Test via a Universal Testing Machine (UTM)

The three-point bending test was used to assess the printed PLAPCL4060, PLAPCL5050, and PLA structures. The means of each sample were obtained by plotting the load vs. displacement (the average mean was calculated; for nonlinear regions, the linear parts were plotted). The flexural modulus for each sample was calculated using Equation (1). The variable values were as follows: support span (L) at 14 mm, width of beam (b) at 3 mm, depth of beam (d) at 2 mm, and slope (m).

(1) $$E_{f}=\frac{L^{3}}{4bd^{3}}$$

**Figure 15:**
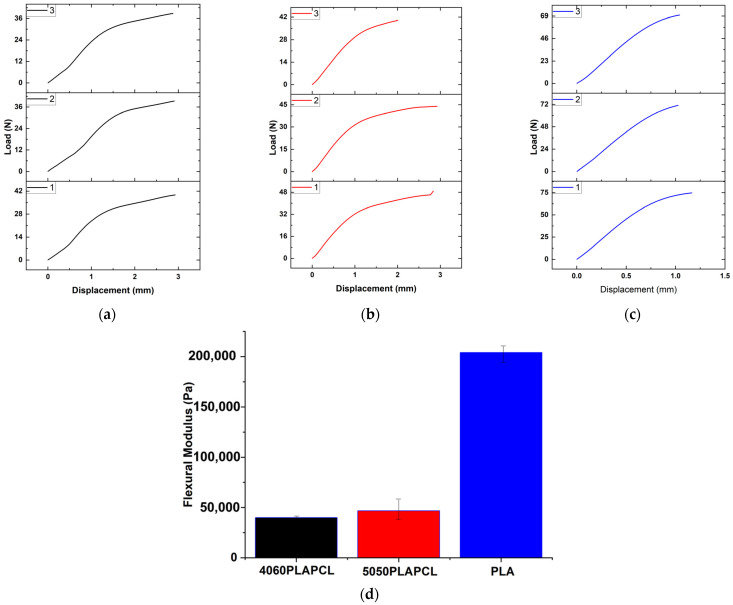
Load vs. displacement plots from the three-point bending test for (**a**) PLAPCL4060, (**b**) PLAPCL5050, and (**c**) PLA printed structures to find the slope. (**d**) Calculated flexural modulus values for PLAPCL4060, PLAPCL5050, and PLA.

The flexural modulus average for each sample was found to be (Figure 15) 39.869 ± 1.52 kPa for PLAPCL4060, 46.705 ± 10.2 kPa for PLAPCL5050, and 203.989 ± 8.75 kPa for PLA. Additionally, due to the limitations of 3D printing with PCL filaments, the three-point bending test analysis did not include PCL samples.

### 3.4. Application of Ultrasound for Bone Tissue Scaffolds

#### 3.4.1. Design and 3D Printing of Triply Periodic Minimal Surface Scaffolds

**Figure 16:**
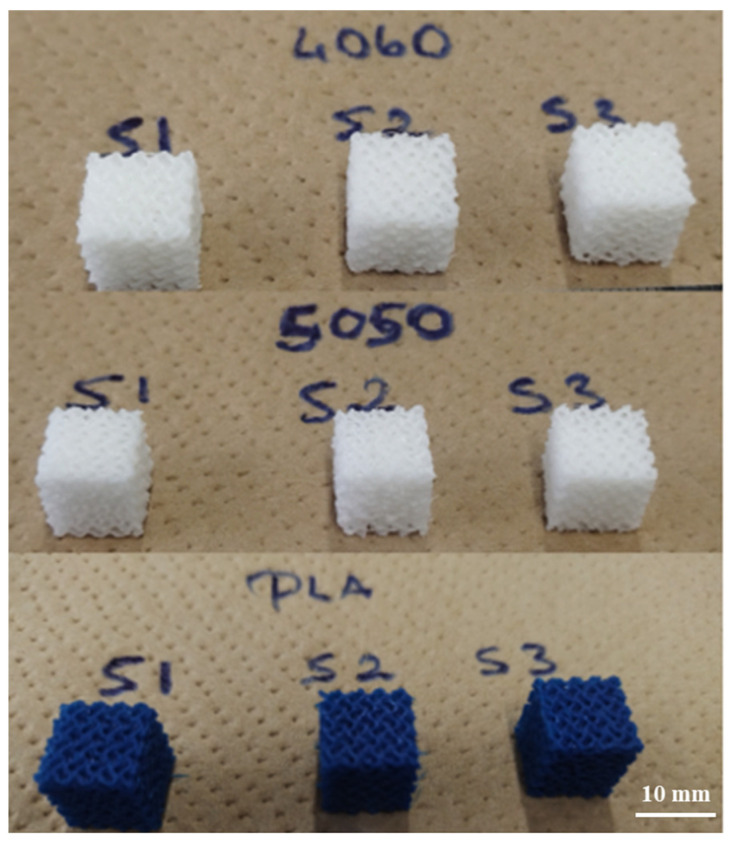
The 3D-printed TPMS scaffolds: (**a**) S1, S2, and S3 (top to bottom); (**b**) PCL scaffold.

The 3D-printed scaffolds of S1, S2, and S3 for PLAPCL4060, PLAPCL5050, and PLA are shown in Figure 16. Due to limitations of PCL FDM filaments, there were major defects when the scaffolds were printed, as shown in Supplementary Figure B. As a result, PCL scaffolds were not included in this experiment.

#### 3.4.2. Accelerated Degradation of Scaffolds Under Ultrasound Stimulation

**Figure 17:**
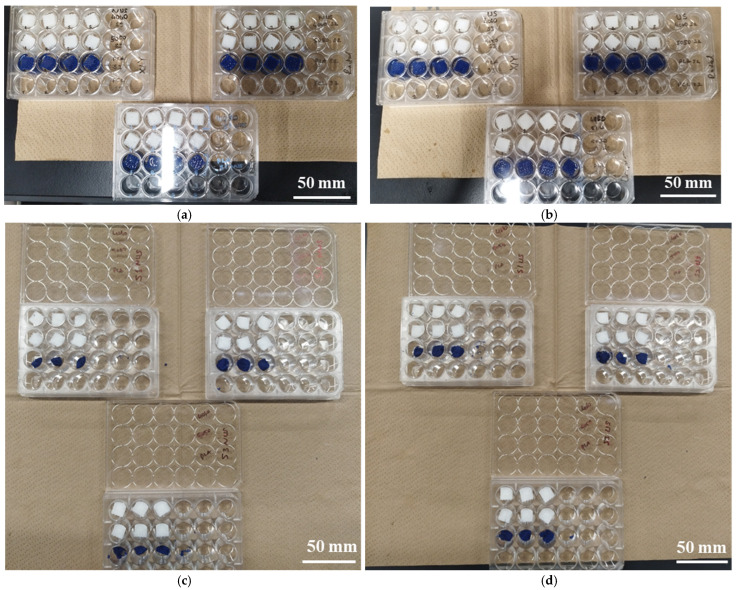
Samples after 1 h of 5 M NaOH: (**a**) NUS S1, S2, and S3 samples of PLAPCL4060, PLAPCL5050, and PLA, and (**b**) US S1, S2, and S3 samples of PLAPCL4060, PLAPCL5050, and PLA (+ 1 h US treatment at RT). Samples after a total of 6 h of incubation (at 37 °C) with 5M NaOH and being dried: (**c**) NUS S1, S2, and S3 samples of PLAPCL4060, PLAPCL5050, and PLA, and (**d**) US S1, S2, and S3 samples of PLAPCL4060, PLAPCL5050, and PLA.

**Figure 18:**
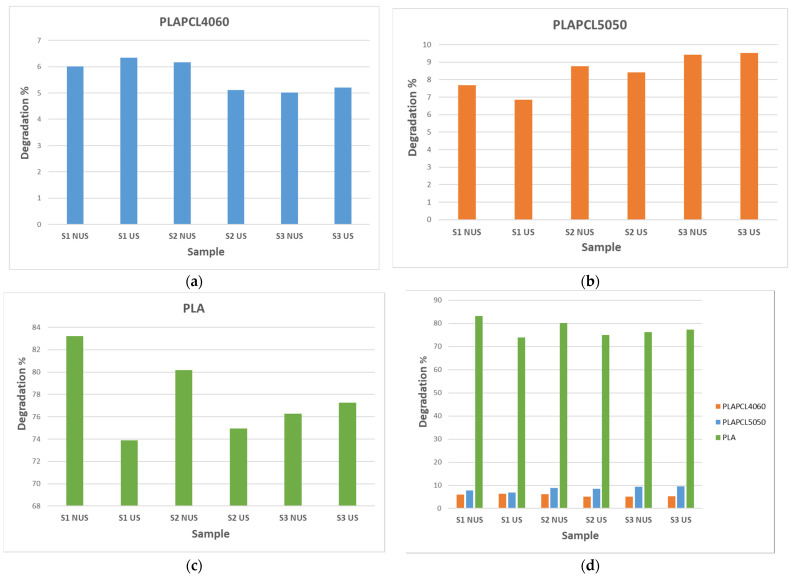
Degradation percentages after a total of 6 h of incubation (at 37 °C) with 5M NaOH (+ ultrasound stimulation for 2h at RT) treatment for scaffolds: (**a**) PLAPCL4060, (**b**) PLAPCL5050, and (**c**) PLA. (**d**) Comparison between PLAPCL4060, PLAPCL5050, and PLA scaffolds (NUS and US).

The results of the accelerated degradation test are given in Figure 17 and Figure 18. The results after 1 h of 5M NaOH being applied to NUS samples incubated at 37 °C (Figure 17a), as well as after 1 h of 5 M NaOH being applied to samples incubated at 37 °C and 1 h of US treatment at RT (Figure 17b), are shown. The results for the NUS samples (c) and US samples (d) at the end of the 6 h experiment are given in Figure 17. The amount of weight lost after 6 h is given in Figure 18. As can be seen in Figure 17, at the end of 6 h of incubation at 37 °C treatment, the PLA scaffolds were completely degraded in both the NUS and US treatments. However, the S1, S2, and S3 PLAPCL4060 and PLAPCL5050 scaffolds were still intact. Yet, the layers can be separated for all these samples. Figure 18 shows the degradation percentages after a total of 6 h of incubation at 37 °C, which were calculated using the difference between the initial and final dry weights of the samples normalized to an initial weight of 100 (((*W_i_* − *W_f_*)/*W_i_*) ∗ 100). As seen in Figure 18a, for the PLAPCL4060 samples, the degradation percentages varied between scaffolds with different gradients. For S1 and S3, the degradation percentage was slightly higher for the US samples than for NUS ones, while for the S2 NUS samples, the degradation percentage was higher compared to the US ones. Comparing the pore gradients based on the degradation percentages, the following outcomes were observed. For PLAPCL4060 NUS samples, it was shown that uniform and radial gradients had a close degradation percentage (6.01% and 6.15%, respectively) and were higher than the x/y gradients (5.011%) (radial% > uniform% > x/y%). For PLAPCL4060 US samples, the uniform porosity degradation percentage (6.34%) was higher than that of both radial and x/y gradients (5.11% and 5.20%, respectively) (uniform > x/y > radial). These results show that, under ultrasound stimulation, pore gradients affect the wave propagation and the degradation kinetics that occur because of stimulation.

For the PLAPCL5050 samples (Figure 18b), the S2 and S3 NUS samples showed higher percentages, while for S1, the opposite trend was observed. Comparing the pore gradients based on the degradation percentages, the following outcomes were observed. For PLAPCL5050 NUS samples, the x/y degradation percentages based on pore geometry were x/y gradients% (9.41%) > radial gradients% (8.75%) > uniform% (7.67%). For US samples, the same behavior was observed (9.52%, 8.40%, and 6.85%, respectively). For PLA samples, the degradation percentage was similar to that of PLAPCL5050 in terms of the S1, S2, and S3 scaffold types for US samples. For the PLA NUS samples, comparing the pore gradients based on the degradation percentages, the trend was uniform (83.92%) > radial (80.17%) > x/y (76.2%). Additionally, for US samples, x/y (77.2%) > radial (74.9%) > uniform (73.8%). However, due to their complete degradation, there was some loss of samples within the solution that should be noted. Furthermore, when compared (Figure 18d), PLAPCL4060 showed a lower percentage compared to PLAPCL5050, with a difference in scaffold types.

#### 3.4.3. Morphological Analysis of Degraded Samples Using Scanning Electron Microscopy

**Figure 19:**
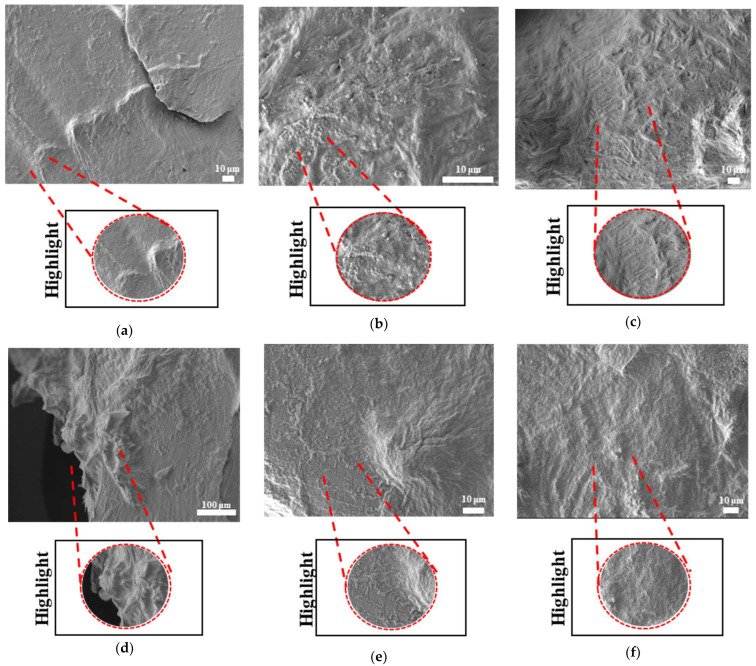

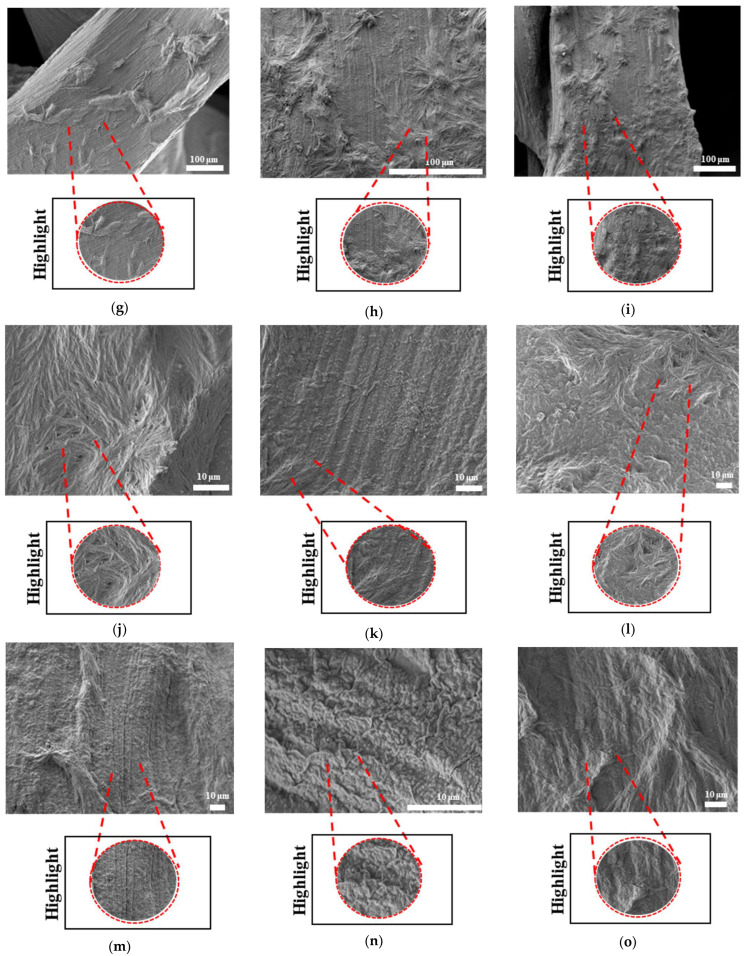

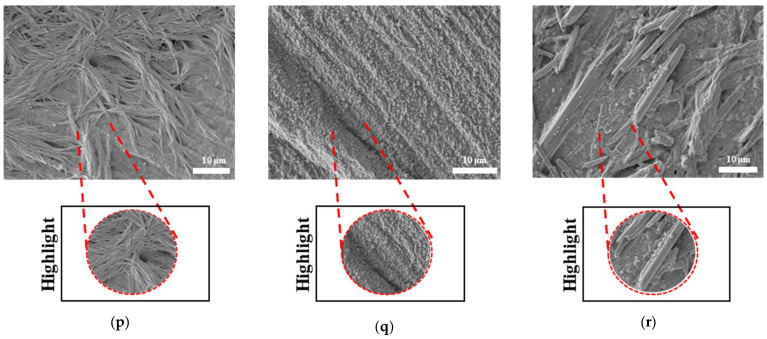
Scanning electron microscopic images of PLAPCL4060 S1, S2, and S3 scaffolds with ultrasound ap-plied (US) and not applied (NUS): (**a**–**c**) S1 NUS, (**d**–**f**) S1 US, (**g**–**i**) S2 NUS, (**j**–**l**) S2 US, (**m**–**o**) S3 NUS, and (**p**–**r**) S3 US.

**Figure 20:**
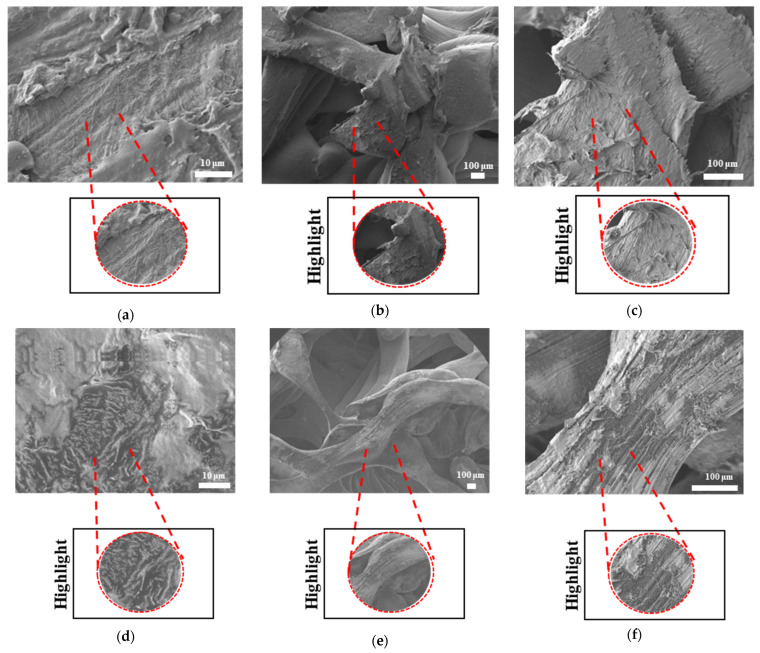

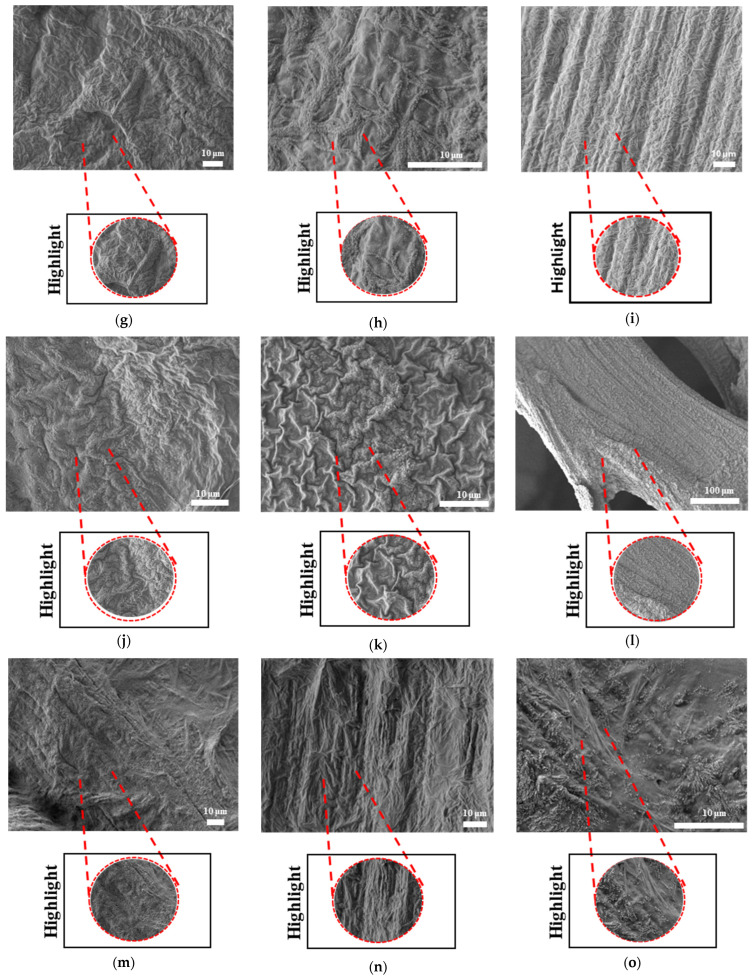

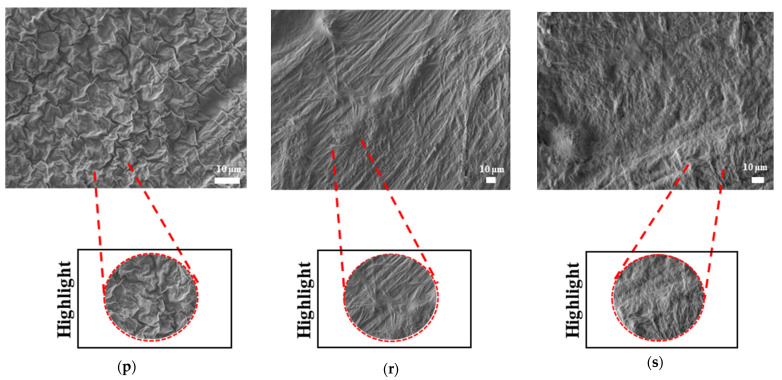
Scanning electron microscopy images of PLAPCL5050 S1, S2, and S3 scaffolds with ultrasound applied (US) and not applied (NUS). (**a**–**c**) S1 NUS, (**d**–**f**) S1 US, (**g**–**i**) S2 NUS, (**j**–**l**) S2 US, (**m**–**o**) S3 NUS, and (**p**–**s**) S3 US.

**Figure 21:**
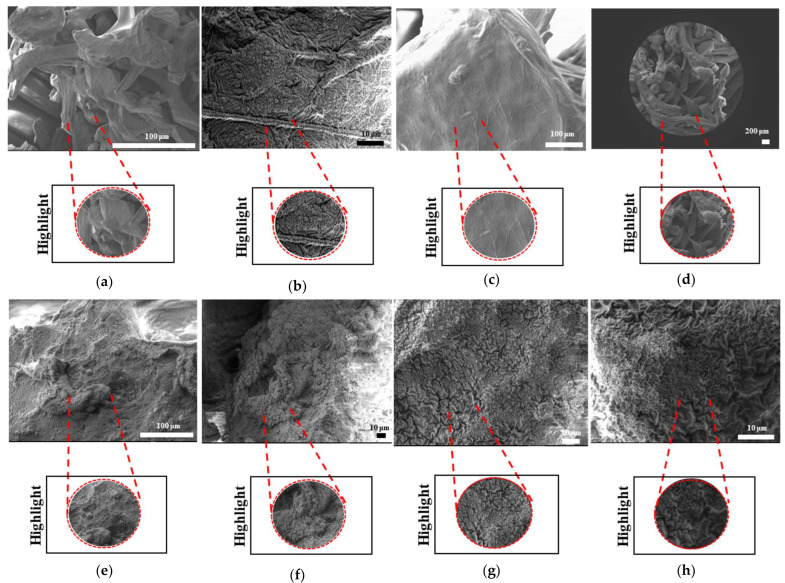

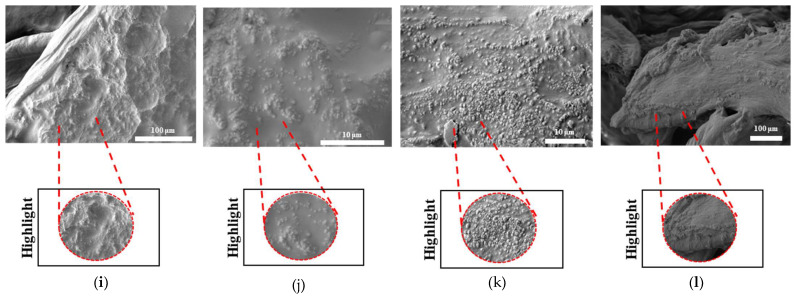
Scanning electron microscopy images of PLA S1, S2, and S3 scaffolds with ultrasound applied (US) and not applied (NUS). (**a**,**b**) S1 NUS, (**c**,**d**) S1 US, (**e**,**f**) S2 NUS, (**g**,**h**) S2 US, (**i**,**j**) S3 NUS, and (**k**,**l**) S3 US.

The SEM analyses of the accelerated degradation of scaffolds under ultrasound stimulation for uniform TPMS (S1), radial TPMS (S2), and x/y gradient scaffolds (S3) are given in Figure 19, Figure 20 and Figure 21. The observations were performed from both the surface and inner part of the scaffolds. Overall, several different degraded surfaces were observed, which are defined here as degradation patterns; each one of the unique patterns is reported in Figure 19, Figure 20 and Figure 21. As seen in Figure 19, for S1, the degradation patterns have similar features from a qualitive perspective. However, when comparing Figure 19c,e, the degradation pattern can be observed even from a larger scale for US scaffolds. For S2, similar string-like degradation patterns were observed in both NUS and US samples, although they could be observed in more detail in the US samples. For S3, US samples showed similar patterns to (m)–(r); however, as seen in t, there was also a difference. In Figure 20, for S1, as seen in (d) and (f) for US scaffolds, the degraded areas show a color difference and defects throughout strut surfaces. For S2 samples, similar patterns were observed, with the exception of (k). For S3, both the NUS and US scaffolds exhibited string-like surfaces, with the exception of (p). The PLA samples are shown in Figure 21; however, due to PLA scaffolds degrading to the point where the scaffold shape was lost, as mentioned in Section 3.4.2, only two images per scaffold and treatment are given. For S1 and S3, similar degraded surfaces were observed between US and NUS samples; however, for S2, US and NUS scaffolds showed different types of degradation patterns.

## 4. Discussion

In this study, the potential of using commercial filaments for ultrasound technology in bone tissue scaffolds was investigated. For this purpose, experiments were conducted, and the results of these experiments are reported herein.

Even though a combination of commercial filaments of PLA and medical-grade polymers of PCL was used for the production of customized filaments, forward characterizations were all conducted using PCL FDM filaments. Such an approach was chosen due to the limitation of not being able to use medical-grade polymers with FDM printers. However, the results and following statements are believed to hold valid reasoning due to both types being the same polymer in different forms (FDM filament and pellets), as well as our main aim being the adaptation of ultrasound technology using FDM printers for accessibility.

The scaffolds displayed in Figure 7 are the outcome of an analysis of the printing conditions. As seen in Figure 7a, the resolution of the printed samples changes for PLA/PCL composite filaments depending on the scale they are printed on. It was found that in comparison to PLA, the FDM filaments of PLAPCL4060 and PLAPCL5050 could not maintain their precision and quality within the overall structure as the complexity increased and dimension size scale decreased. Furthermore, a comparison of two PLAPCL mixture-based constructions revealed variations in quality and precision, as shown in Figure 7a,c. PLAPCL5050 samples provided continued precision, while PLAPCL4060 scaffolds showed defects in patterns as the structure became complex (Figure 7a,c). Such an observation can be explained by the higher percentage of PLA in PLAPCL5050 scaffolds. PLA, with its lower flexibility, is easy to adapt for use in various printing methods, whereas PCL has been known to be challenging, especially in FDM printing.

Pure PCL shows peaks at 2θ = 21.5° and 23.85°, corresponding to the crystal planes of (110) and (200), respectively [49]. However, in this study, XRD spectra (Figure 8) revealed shifted peaks for PCL FDM filaments and customized filaments containing PCL (PLAPCL4060 and PLAPCL500) for both printed structures and filaments alone. Such phenomena could be explained in several ways. One of the reasons could be the presence of additional compounds in FDM filaments causing the change within the matrix, as seen from Raman spectra identification for certain samples (see Supplementary Materials, Supplementary Raman) that were different from the samples’ original polymer. For instance, Zhou et al.’s XRD and Raman spectra results for pure PLA display peaks that are different from those in Figure 8 and Figure 9 [50]. These results, as well as those of several other studies, provide evidence that can be used to support the idea that the shifts potentially occur due to the commercial nature of the filaments [51,52]. These results can also be explained by focusing on the XRD spectra results for PLAPCL4060 and PLAPCL5050. It is possible that chain confinement during PCL crystal growth or the solid PLA served as a nucleation site for PCL crystal formation during PCL crystallization in a PLA/PCL blend. Sanandaji et al. [53] assert that substantial chain confinement results in elevated equilibrium melting temperatures, reduced melting temperatures (Tm), and diminished crystallinities. No significant alterations in T_m_ were noted between PCL and PCL in the various blends (Table 1); however, the normalized crystallinity of PCL in Table 1 diminished as the PCL fraction in the blends decreased. In addition, the T_eq_ m values for PCL in the blends were marginally elevated compared to those of neat PCL (Table 2). These two discoveries may suggest chain confinement in the blends when PLA constitutes the predominant phase, which would also explain the tighter crystal size distribution for PCL in the blends [54]. Another reason behind the shifts could be due to the crystallinity changes that occur within the polymer during the extrusion process. The crystallinity of polymers can be adjusted in various ways. One of these ways is to introduce additives to the polymer matrix through physical treatment (e.g., solution mixing, evaporation, and annealing) [55]. It is possible that during the extrusion process, the additives present in the PLA FDM filament might have caused the change in crystallinity. Another cause of the potential shifts can be explained from the basic description of the occurrence of crystallinity within polymers. In the literature, it is known that upon cooling from melting, solvent evaporation, or mechanical stretching, polymers can crystallize. Therefore, even without the additive, the heat treatment that occurs during extrusion might be one of the reasons that causes the shifts in crystallinity peaks.

The Raman spectra identification results (Figure 9) based on the referenced database used in Origin (Figure 9), as well as the Raman spectra database provided within open-source software (OpenSpecy) (see Supplementary Materials, Supplementary Raman), have provided information supporting the XRD results and the potential shifts that were observed in the XRD spectra. Our Raman spectra analysis (Figure 9) showed the occurrence of phenomena similar to those observed in the XRD results; i.e., there were shifts from the referenced Raman shifts for PLAPC4060 and PLAPCL5050, which matched with the reference PLA and PCL Raman shifts. Such changes could be due to the presence of additives that were used during the production of commercial FDM filaments. However, as shown in Section 2.3.4., for both filament and printed samples, Raman shifts regarding the databases of PLA and PCL matched exactly with the referenced literature. As such, from then on, any deductions were performed while considering the shift changes that were observed. For PLAPCL4060, the presence of PCL and PLA was detected both in the filament and printed samples, while in PLAPCL5050, only PLA peaks were detected. Such outcomes show a divergence from the results provided in the Supplementary Materials, which were based on the OpenSpecy spectrometer.

The Raman spectrum analysis shown in Supplementary Raman suggests the presence of PCL in filaments of PCL and PLAPCL4060, as well as in the printed structures of PLAPCL4060, PLAPCL5050, and PCL. However, for PLA and PLAPCL5050 filaments and printed PLA structures, the identification did not show the presence of either PLA or PCL. As mentioned in the previous paragraph, these deviations from the original polymer could be due to additional materials being used during the production of commercial filaments. However, such outcomes do not diminish the potential of commercial filaments for use in ultrasound technology. Moreover, the presence of PCL provides proof of a successful integration between commercial and medical-grade polymers for use in FDM printing.

Based on the XRD and Raman spectra analysis, a further look at the ultrasound responsiveness of these filaments and the implications of these potential causes (e.g., additive, chain confinement, and crystallinity change) should be discussed in detail. The presence of additives as a result of FDM filaments, in relation to ultrasound responsiveness, could be discussed from a polymer matrix perspective. From a basic definition of ultrasound propagation, the physical properties of the medium (acoustic velocity, density, elasticity, and impedance) affect the propagation of ultrasound waves. Thus, in the case of the additive’s presence in a matrix, the density of sections that have additives might potentially differ; the elastic modulus could potentially be affected, as well as the impedance. As the density of the medium increases, the acoustic impedance will increase. Similarly to the difference between a soft tissue like the lung and a hard tissue like bone, the large acoustic difference will create a strong reflection. From an ultrasound responsiveness point of view, one can deduce that a strong reflection would decrease its responsiveness since the reflected wave would not be able to reach deeper layers. Additionally, only a small number would be traveling to deeper layers. However, this issue could be easily resolved by further investigating the correlation between height and penetration depth in order to define a range of dimensions for bone tissue scaffolds. There is a potential for chain confinement in the polymer matrix due to diminished crystallinities; such cases can affect the responsiveness of the matrix. Based on the reference article, the responsiveness of PLA/PCL comes from the PLA acting as a reflecting element (amorphous from melt) and PCL (crystalline from melt) as a damping element within the PLAPCL matrix [24]. However, in theory, the crystallinity changes could potentially be enhanced (in the case of additional crystallinity formations) or decreased (in case the amorphous nature of PLA is affected). Thus, all of the potential causes of the observed shifts in peaks can potentially affect the ultrasound responsiveness in both a positive and a negative way.

Scanning electron microscopy was primarily used to investigate phase segregation. For such a purpose, the contrast differences mentioned in [24] and the surface structures were taken into account (for the presence of crystal and amorphous structures). The results from Figure 10c show a contrast phase difference between lighter gray (PLA, according to the reference article, is observed to be bright) and darker gray (PCL, according to the reference article, is observed to be dark) for PLAPCL4060. For printed PLAPCL4060 (Figure 11), the same difference can be observed, accompanied by nonhomogeneous surface structures. However, for PLAPCL5050, no such difference in the means of contrast was observed, even though a variance in the surface structures was present. Such results led to the conclusion that a phase segregation for PLAPCL4060 commercial filaments was achieved, differing from the 50:50 PLA:PCL ratio reference articles. In the referenced study, polymers with equal MWs were used, while FDM filaments are reported to be known to potentially have a MW range (PLA: 50,000–140,00 Da, containing the 60,000–80,000 Da); as such, differences in percentages were expected. We would like to mention that even though contrast differences were observed, the limitations of the methods and the results should be discussed. Even though qualitative evidence of the contrast difference was observed, there are still limitations that can potentially arise from using SEM as a method of investigation. As reported in the literature, SEM only allows for a limited amount of thickness to be observed and does not provide a characterization of bulk samples. However, due to the homogeneity of the sample composition and the uniformity between samples prepared for SEM observation, one can take such observations as representative of the bulk structure. Another limitation comes from SEM being based on the main principle of electron microscopy. The reflecting electrons are the ones that create the image, and they can be affected by any height differences that exist on the sample surface. However, the samples used for these experiments (Figure 11, Figure 12 and Figure 13) were all the same scaffold structures that possess the exact same patterns and geometry. Thus, such uniformity prevents any limitation that can affect the outcomes. On the other hand, we also acknowledge the limitation that comes from SEM’s surface-specific nature, which can be summarized as follows. SEM mostly offers details about a sample’s outermost layer. This restriction results from the method’s operation, whereby an electron beam scans the surface, and the image that is produced depends on how the electrons interact with the material. SEM can show the composition and surface morphology in detail, but it cannot reveal the sample’s bulk characteristics or deeper structures. Furthermore, a more detailed investigation with transmission electron microscopy (TEM) can further prove the presence of phase segregation between PLA and PCL. These outcomes and future results (provided from the plans of a detailed investigation with TEM) would further support our main aim of utilizing commercial filaments and FDM technology for ultrasound technology and bone tissue scaffolds.

The nanoindentation results presented outcomes that were consistent with the nature of PLA and PCL polymers (i.e., PCL has flexibility), while PLA has tougher characteristics [56,57]. As for the statistical analysis (Figure 14, there were statistically significant differences between PLAPCL5050 and PLA. Additionally, as seen in Figure 14a–d, as the PLA percentage increased within the filament matrix, both the hardness and elastic modulus increased, while the opposite was observed as the PCL percentage increased. Such outcomes support the nature of PLA and PCL polymers. Furthermore, in terms of the results, Grigora et al.’s study presented a nanoindentation value for PLA filament-based samples of 3945.33 ± 134.74 MPa [58], while our calculations led to a value of 4406.49 MPa. For PCL, PLAPCL4060, and PLAPCL5050, no research based on FDM filaments was found. As shown in Table 2 and Table 3, no significant difference was found between PLAPCL4060 and PLAPCL5050. This result could be due to only a 10% difference being used in the mixtures. Further investigation with a greater range of PLA and PCL percentages would provide information regarding how individual PLA and PCL characteristics affect the mechanical characteristics of FDM filaments. However, these non-significant differences can also be seen as an advantageous property when taking into account that PCL reduces brittleness and PLA increases the elastic modulus. Because of its moderate tensile strength, better elongation at break when compared to pure PLA, and increased toughness from the addition of PCL, this FDM filament is a good option for applications that require flexibility and impact resistance while still retaining some biodegradability. For bone tissue scaffolds, these characteristics are important, since bone tissue is exposed to daily forces that require endurance and long-term stability. Since PLAPCL4060 and PLAPCL5050 show superior mechanical characteristics compared to PLA in terms of elasticity and brittleness, depending on the purpose of the application, either of the FDM filaments can be used. On the other hand, a lower elastic modulus leads to application limitations. This can be improved by introducing an element to the matrix that can support the load distribution and increase the elastic modulus, in a similar manner to how researchers introduce electro-spun fibers to hydrogel matrixes. Furthermore, depending on the scaffold and porosity geometry, the mechanical strength can be adjusted. There are various studies in the literature that focus on PLA-PCL composite filaments and their mechanical characteristics [59,60,61]. One study in particular involves percentages of PCL60PLA40 (wt/wt) and PCL50PLA50 (wt/wt), similarly to our work. Haq et al. investigated the elastic modulus of FDM filaments based on various combinations of PLA-PCL biopolymers using tensile testing [62]. Based on this study, one can deduce that there was a significant difference between the PCL60PLA40 and PCL50PLA50 samples, which contradicts our mechanical testing. However, in that study, since the researchers used tensile testing and, in our study, nanoindentation was used, one should pay attention to the significant difference between how they are conducted and what they provide in terms of mechanical characterization. Nanoindentation facilitates the precise characterization of the mechanical properties at the nanoscale; the process of applying a tiny, focused force to the surface of a material; and evaluating the resulting indentation depth and load–displacement behavior, which is particularly important for materials that are composed of more than one component. On the other hand, tensile testing is determined by stretching the material under controlled conditions. Furthermore, we would also like to draw attention to printing conditions as one of the potential reasons for the observed non-significant difference. Xiao et al. investigated the influence of specific 3D printing parameters, including printer head temperature, fiber diameter, and degradation, in relation to the mechanical characteristics of FDM-printed polyethylene materials. As a result, they have observed that Young’s modulus and tensile strength decrease as the printer head temperature increases, while smaller-diameter FDM-printed PE fibers that are created at a higher collection speed experience greater stretching, which raises their tensile strength and Young’s modulus values [63]. Thus, we believe that the FDM printing conditions might be another explanation. As an aside, we would like to point out the differences between each filament and explain the reasoning behind them in order to prevent any further misunderstandings. During nanoindentation testing, there were points that did not have contact with the sample’s surface due to the last layer of the STL-contained structure. As a result, these were not taken into consideration. In addition, if the samples moved during or immediately after testing, they were also not included in order to prevent false results. Also, as seen in Figure 14, the standard deviation (error bar) for PLA was higher compared to that of PLAPCL4060 and PLAPCL5050. This was observed due to the presence of a very low value observed from one of the indentation points. Since the non-contact point was not an error in this case, the value was still included within the calculations. Despite these problems, the results of the statistical analysis showed a logical correlation between each polymer and polymer percentage. Furthermore, as explained in Section 3.3.4, due to the limitations of PCL filaments, PCL was not included in our discussion.

Furthermore, when discussing the lack of significant difference between the elastic modulus of PLAPCL4060 and PLAPCL5050, one should also consider its effect on the flexibility properties of scaffolds produced from these polymer blends. As discussed previously, as the PLA percentage increased within the filament matrix, the elastic modulus increased, while the opposite was observed as the PCL percentage increased. Additionally, it is known that the elastic modulus and flexibility of a material have a reverse correlation. Therefore, one can deduce that as the PLA percentage increases, the flexibility of the scaffold under ultrasound stimulation will decrease. However, in our case, since we have not observed a significant difference, both PLAPCL4060 and PLAPCL5050 should be considered as options for ultrasound application.

The flexural modulus results showed consistency with the nanoindentation characterization results. However, the results for the elastic and flexural moduli were different for the samples with the same dimensions, instead matching with the nature of polymeric materials. Additionally, in PLA vs. PLA/PCL, PLA was found to be approximately 3–4 times greater than PLAPCL5050 and PLACPL4060, which is a result similar to that seen in [24]. This analysis indicates that PLAPCL4060 and PLAPCL5050 have better flexibility compared to PLA, which should be considered when designing a scaffold that requires responsiveness and dynamicity under ultrasound exposure.

With an aim to investigate the potential of commercial FDM filaments for ultrasound technology in bone tissue scaffolds, characterizations using XRD, SEM, Raman spectra, and three-point bending were conducted in relation to scaffold composition, mechanical characteristics, and printability. In light of these characterizations of composite filaments based on PLA and PCL for their potential in ultrasound technology, one can deduce their alignment and importance to bone tissue scaffolds. From a compositional perspective, the biomaterials used in bone tissue scaffolds have been chosen by researchers depending on their purposes, such as for enhanced physical and chemical properties, stimuli responsiveness, or enhanced mechanical properties [64,65,66]. In this study, PLA and PCL biopolymers were used with the purpose of creating a dynamic scaffold that responds to ultrasound stimulation. One of the most promising combinations for tissue engineering is known to be PLA/PCL combinations. The key features of these polymeric compounds are their high purity, suitability for processing, and exceptional mechanical properties, which have enabled the development of scaffolds with a wide range of applications in regenerative medicine. Furthermore, it should be noted that these materials are biodegradable and that their breakdown products can be reabsorbed. As a small organic molecule, lactic acid has a variety of physiological and metabolic routes. These two chemicals improve the biomechanical performance of the constructs, and some studies have found benefits in both mechanical and biological properties [67]. The XRD and Raman spectroscopy results showed the presence of these two polymers (with shifts from the reference values) in PLAPCL4060 FDM filaments. The potential reasons for the shifts have already been presented and discussed. Furthermore, from a bone tissue scaffold perspective, any limitations could be caused by impurities in the commercial filaments, which are believed to have affected the results obtained. PLA FDM filaments contain a number of additives, e.g., coupling agents, impact modifiers, processing aids, nucleating agent, antioxidants, colorants, and flame delayers. These impurities are used during manufacturing to achieve high-performing PLA FDM filaments; however, these additives can be disadvantageous in bone tissue scaffold scenarios. In contrast, it is known that the presence of some additives can also improve the mechanical properties. However, they can also cause decreased adhesion between printed layers. As a result, one can deduce that the presence of additives can be both advantageous and disadvantageous. Additionally, from a clinical application perspective, despite the abovementioned advantageous properties of PLA/PCL combinations, the presence of additives in FDM filaments can limit their application. In particular, the cytotoxic fumes that are produced during the printing process have been investigated in the literature with various types of cells. However, we would like to point out that these studies focus on the ultrafine and fine particles that are emitted during the manufacturing process [68,69] or during the sterilization process [70]. Additionally, there are studies which use commercial FDM filaments with an aim to assess their biocompatibility. Shilov et al. used 3D-printed PLA, PEEK, and PETG to assess the relation between cell adhesion and printing parameters. As a result of the cytocompatibility test they conducted, no cytotoxic effect was reported [71]. However, we would like to address the potential cytotoxicity based on the study of Burkhardt et al. In this study, researchers investigated the cytotoxicity of polypropylene (PP) and biocopolyester (BE), which are utilized to fabricate surgical guides using fused filaments. As a result, they came to the conclusion that all materials are biocompatible in vitro in the short term, while in the long term, they expressed potential inflammatory response [72]. In light of these studies, we acknowledge the potential cytotoxicity risk that can potentially occur in long-term applications as a result of the aforementioned additives. To ensure their adaptation to clinical applications and to investigate the potential cytotoxicity, in vitro tests have been planned for future part of this investigation. Briefly, the in vitro tests can be outlined as follows. By using the MC3T3-E1 Subclone 4 cell line with the minimum essential medium—Alpha Modification (Alpha-MEM)—as a growth medium, supplemented with 10% fetal bovine serum and 1% penicillin–streptomycin solution, experimental optimization will be conducted to assess the cell number and mineralization medium. For this purpose, (3-(4,5-dimethylthiazol-2-yl)-5-(3-carboxymethoxyphenyl)-2-(4-sulfophenyl)-2H-tetrazolium) MTS, ALP, and Alizarin Red staining assays will be conducted. After the optimization studies, cell viability (MTS), differentiation (ALP and Alizarin Red S Staining), and immunocytochemistry with Phalloidin staining will be conducted [73]. These assays will provide the information required for transitioning from its current lab-scale status to clinical applications, while addressing the potential cytotoxic nature of commercial FDM filament-based scaffolds.

From the perspective of mechanical characteristics, in this study, three mechanical characteristics—flexural modulus, hardness, and elastic modulus—were investigated. For a bone tissue scaffold that responds to ultrasound stimulation, the flexural modulus provides important information. Based on the flexural modulus, one can deduce the stiffness and the resistance to bending under load. A lower flexural modulus means that the material is more flexible, correlating with the ability of the scaffold matrix to change its shape under load, which, in this study, results from ultrasound stimulation. In terms of future clinical applications, since there are applications of ultrasound that have already been established and used in clinics, it can be said that the potential transition from research to clinics is highly likely. The elastic modulus and hardness results for PLAPCL4060 and PLAPCL5050 showed no significant differences; therefore, in terms of bone tissue scaffold applications, such a result could potentially open future opportunities based on the aims of the researchers. Additionally, as previously mentioned, the mechanical characteristics can be adjusted depending on the geometrical design and the porosity specifications.

From a printability perspective, in this study, scaffolds with complex geometrical structures were printed on 200% and 125% scales in order to assess the limitations and the printing parameters. Porosity percentage, pore size, pore geometry, and overall scaffold geometry are crucial properties when designing bone tissue scaffolds. Due to bone tissue’s complex geometrical structure and hierarchy, designing and manufacturing bone tissue scaffolds has been part of bone tissue engineering for years. The results for PLAPCL4060 and PLAPCL5050 show that there were indeed some limitations for PLAPCL4060 FDM filaments, since some defects within the scaffold pattern and geometry were observed. This will limit the design range of bone tissue scaffolds, as well as their potential clinical applications; however, the FDM printer quality also plays a role. Therefore, using an FDM printer with higher specifications and precision could potentially be a solution to this problem.

As previously stated, researchers have recently investigated the potential of utilizing ultrasound stimulation (US) in conjunction with tissue scaffolds to enhance tissue growth and regeneration. To further investigate the potential of PLAPCL4060 and PLAPCL5050 for ultrasound technology in bone tissue scaffolds, accelerated degradation experiments for TPMS structures with varying porosity gradients were conducted. We utilized NaOH degradation to understand time- and composition-related degradation mechanics, which are important factors in bone tissue scaffolds in the context of healing processes. For bone tissue scaffolds, as the defective tissue regenerates, the scaffold in question should degrade, allowing new tissue formation to replace the scaffold. Therefore, this section of this study focused on such applications by investigating the weight change and surface morphology. However, we would like to point out that the degradation observed in this study has been in the form of mechanical degradation, rather than polymeric degradation. Furthermore, we have discussed the effect of this mechanical degradation, as well as the patterns that were observed as a result of this. Additionally, when investigating degradation, it was determined that the rate of degradation depends on the initial state of the polymer; thus, parameters such as molecular weight and crystallinity are important. In the literature, our initial investigation of an FDM filament’s molecular weight resulted in observing a wide range, depending on the manufacturer’s intended aim. However, as the investigation continued, as well as a general range of 60,000–80,000 Da MW, we also observed a range of 50,000–140,000 Da MW being discussed in several articles [74,75,76]. This is a common issue when working with commercial FDM filaments. The technical datasheet includes information regarding density and temperature, but lacks information such as molecular weight and crystallinity. However, to improve the accessibility of the FDM filaments, a future study has been planned. In the future study, it is planned to investigate the MW, crystallinity of PLA FDM filaments, and crystallinity of PCL medical-grade pellets in order to better understand the degradation kinematics and also investigate the degradation in the long term in order to achieve polymeric degradation.

As shown in Figure 18, despite the small and non-significant differences between PLAPCL4060 and PLAPCL5050, there was a variation between scaffold types and between the FDM filaments that the samples were produced from. Briefly, the weight differences before and after a total of 6 h incubation at 37 °C with 5 M NaOH (+ 2 h RT ultrasound stimulation for US samples) treatment were as follows: (1) PLA scaffolds degraded independently of scaffold type and the presence of ultrasound stimulation. Between scaffold types S1 and S2, the sample degradation percentage was higher for NUS samples; for S3 scaffolds, it was the opposite. (2) PLAPCL4060 S1 scaffolds and PLAPCL5050 S1 scaffolds showed contrasting responses to ultrasound stimulation. From these results, one can deduce that, for uniform gyroid bone tissue scaffolds (S1), ultrasound stimulation can accelerate the degradation process in FDM filaments with ultrasound-responsive characteristics. (3) For S2 and S3 scaffolds, the beforementioned composite FDM filaments showed a similar behavior within the scaffold types. For radial gradient gyroid bone tissue scaffolds (S2), independently from possessing ultrasound stimulation responsiveness, ultrasound stimulation seemed to decelerate the degradation. Such an outcome could be either due to the presence of ultrasound stimulation or due to the radial gradient geometry that the scaffolds possess. For x/y gradient gyroid bone tissue scaffolds, the opposite was observed compared to S2 scaffolds, whereby the presence of ultrasound stimulation accelerated the degradation process. (4) Despite their complete degradation and the potential errors that can occur, when compared to PLAPCL4060 and PLAPCL5050, the results from the PLA scaffold provide the following statements.

Considering the outcomes from FDM filament characterizations, the PLAPCL4060 FDM filament was claimed to possess ultrasound-responsive characteristics; in the context of accelerated degradation, a deduction can be performed for uniform gyroid scaffolds. When PLA S1 scaffolds are compared to PLAPCL4060 and PLAPCL5050 scaffolds, it is seen that US samples accelerated the degradation of the scaffolds produced from ultrasound-responsive FDM filaments, while for the ones that do not have such characteristics, the results were the opposite. Furthermore, when compared to the porosity gradient type and their responsiveness to ultrasound stimulation, it can be deduced that radial gyroid gradients affect the ultrasound wave propagation and degradation kinetics, which could be seen as a limiting factor or a further improvement depending on the aim of the application that one considers. When comparing uniform (PLAPCL4060 US) vs. radial (PLAPCL4060 US) samples, it can be deduced that the pore geometry is one of the parameters that affects the wave prorogation in a tissue scaffold. To further assess pore geometry vs. ultrasound wave propagation, an investigation based on Ferguson et al.’s approach to ultrasonic wave prorogation in randomly layered heterogonies media will be conducted [77]. Additionally, future studies individually investigating these scaffold types to assess their potential in bone tissue scaffolds, such as their biodegradation in phosphate-buffered solution (PBS) or their bioactivity in stimulated body fluid (SBF), would provide a greater understanding.

The surface morphologies of uniform TPMS (S1), radial TPMS (S2), and x/y gradient (S3) scaffolds degrading under ultrasound stimulation were analyzed. Overall, a number of degradation patterns were observed and are reported in Figure 19, Figure 20 and Figure 21. Comparing the results with the degradation percentages shown in Figure 18 for PLAPCL4060 TPMS, Figure 19 S1 NUS (a–c) and Figure 19 US (d–f) aligned with the result of Figure 18a, since the degradation pattern was able to be observed on a larger scale. Figure 19 S3 NUS (m–o) and Figure 19 S3 US (p–t) aligned with the result of Figure 18a. For PLAPCL5050 TPMS scaffolds, the SEM analysis showed that S2 and S3 scaffolds, for both NUS and US treatments, possess string-like degradation patterns, while both had an exception—(k) for the NUS sample and (p) for the US sample. When compared to the degradation percentage results for PLAPCL5050 (Figure 18b), S2 NUS and S3 US were found to be higher. Even though such different degradation patterns do not exactly reflect the degradation percentage difference between these US and NUS samples, it is still a factor that should be considered as part of the supporting conclusions. Furthermore, despite losing their scaffold shape after degradation, PLA scaffolds still show a different degradation pattern for S2 and S3 scaffolds. To further evaluate these outcomes, a literature review found that there are a number of studies that focused on PLA, PCL, and PLA-PCL. However, these studies did not provide information regarding the ultrasound application or the differences in application time and solution concentration used during the experiments. Therefore, no further comparison was conducted.

Overall, our findings support the potential use of commercial filaments for ultrasound technology in bone tissue scaffolds. Based on our aims, the production of PLAPCL4060 and PLAPCL5050 was successful, and the printing condition analysis showed that these filaments could be used in complex tissue scaffold geometries, even with the limitations that come with FDM printing; the search for phase segregation and the presence of both polymers in printed scaffolds was successful; the nanoindentation and three-point bending testing provided the mechanical characteristics for PLAPCL4060 and PLAPCL5050, and presented FDM filaments that have both flexibility and strength for bone tissue scaffolds; and the accelerated degradation provided insights into the ultrasound stimulations of the FDM filaments, as well as into the presence of porosity gradients and their interaction with ultrasound stimulation.

## 5. Conclusions

In conclusion, this study aimed to investigate the potential of using commercial FDM filaments for ultrasound technology in bone tissue scaffolds. According to our objective, commercial FDM filament-based PLA-PCL FDM filaments (PLAPCL4060 and PLAPCL5050) were investigated using various tests, and the results showed promising outputs for their potential as a component in ultrasound technology in bone tissue scaffolds. The successful production of ultrasound-responsive FDM filaments and complex tissue scaffolds was achieved, which improves accessibility for future studies due to the proposed cost-effective and easy-to-adapt method for ultrasound technology applications relating to bone tissue scaffolds. Mechanical characteristics that are favorable for ultrasound stimulation were observed, which further supported the aim of this study. Our accelerated degradation experiments provide interesting insights into achieving enhanced tissue growth and regeneration via the various types of degradation patterns that were determined, indicating how complex tissue scaffolds may respond to ultrasound stimulation. For further transitioning from lab-scale FDM printing to clinical applications, the following steps are planned to be taken as part of future studies.

1. Primary step (lab-scale): Additional investigations into phase segregation, the quality control of FDM-printed samples, and scaffold geometry limitations (e.g., scaffold dimensions, pore geometry, and gradients).

Also, investigations are planned to assess the stability and biocompatibility of the scaffold’s in vitro experiments, such as cytotoxicity and proliferation assays. Furthermore, investigations into the sterilization method, such as gamma irradiation, ethylene oxide, and electron beam sterilization, will be conducted.

2. Secondary step (pre-clinical/pilot scale): Animal testing will be performed to further investigate their safety and efficacy for bone tissue regeneration.

In addition, investigations into the scalability of lab-scale FDM printers will be conducted, as a means of defining the production limits. The production limits we plan to investigate are the number of scaffolds produced in a month, a quality check of each scaffold, and the production cost (chemicals and energy).

Additionally, investigations and adjustment of the ultrasound stimulation set-up will be explored, researching their adaptability to currently used ultrasound technologies in clinical stages.

3. Tertiary step (clinical trial/pivotal scale): Clinical trials (Phase I, Phase II, and Phase III) will be conducted and regulatory approvals (FDA and EMA) will be applied.

Also, the adaptation of manufacturing to large-scale production, as well as considerations relating to quality and distribution to clinics, will be investigated.

In conclusion, future studies focusing on additional investigations of phase segregation and in vitro experiments with ultrasound stimulation and the transition from the lab scale to clinics will further contribute to the use of commercial filaments in ultrasound technology.

## Figures and Tables

**Table 1:** Printing conditions used for PLAPCL4060 and PLAPCL5050 FDM filaments.

Parameters	Value
Scale	125%
Printing Temperature	200 °C
Infill speed	10.0 mm/s
Infill density	15%
Printing Speed	50.0 mm/s

**Table 2:** Tukey’s test results with significant values for hardness.

Sample Hardness	Mean Diff.	SEM	q Value	Probability	Significance	Standard Deviation	SE of Mean
PLAPCL5050 vs. PLAPCL4060	9.056	8.88	1.44	0.738	0	9.24428	1.71662
PLA vs. PLAPCL4060	149.35	9.12	23.15	<0.0001	1	15.69839	1.93234
PLA vs. PLAPCL5050	140.30	7.244	27.39	<0.001	1	74.9949	10.02161

**Table 3:** Tukey’s test results with significance values for elastic modulus.

Sample Elastic Modulus	Mean Diff.	SEM	q Value	Probability	Significance	Standard Deviation	SE of Mean
PLAPCL5050 vs. PLAPCL4060	143.70	168.98	1.20	0.83	0	117.66122	21.84914
PLA vs. PLAPCL4060	3758.18	173.49	30.63	<0.0001	1	214.65722	26.42248
PLA vs. PLAPCL5050	3614.48	137.78	37.10	<0.001	1	1449.70012	193.72433

## Data Availability

The data presented in this study are available from the corresponding author upon request. The data are not publicly available due to data is part of corresponding authors doctoral thesis and on going studies.

## Supplementary Materials

The following supporting information can be downloaded at: https://www.mdpi.com/article/10.3390/bioengineering12050529/s1.
