# Supplementary material for: Pioneering Soundscapes: Investigating Commercial Fused Deposition Modelling Filament’s Potential for Ultrasound Technology in Bone Tissue Scaffolds
Source: Bioengineering (Basel). 2025 May 15;12(5):529. doi: 10.3390/bioengineering12050529 (PMC12108655; doi:10.3390/bioengineering12050529)
Supplement: Supplementary file 1 [file bioengineering-12-00529-s001.zip › Supplementary Raman.pdf]

Supplemental Document

Raman spectra analysis via OpenSpecy software with detected and matched materials are shown in Figure 6. For filaments the matches that detected were as follows: PCLPLA4060 matched with PCL from Sigma-Aldrich, PCLPLA5050 matched with polyester based on the referenced spectra data, PLA matched with poly(vinyl chloride) (pvc) and PCL matched with PCL . For printed structures Raman spectra were matched with as follows: PLAPCL4060 matched with PCL reference spectra, PLAPCL5050 matched with PCL , PLA matched with PVC and PCL matched with PCL from referenced database .

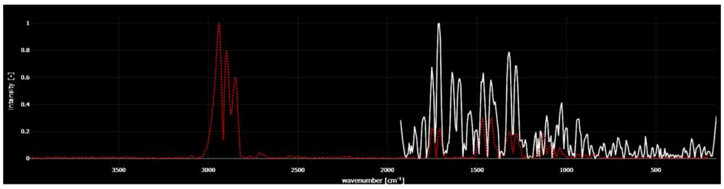

(a)

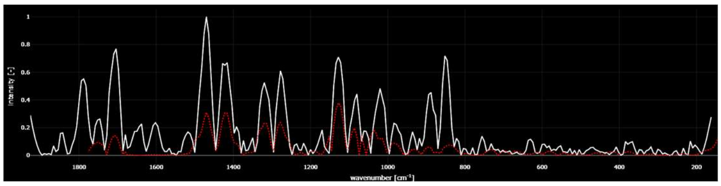

(b)

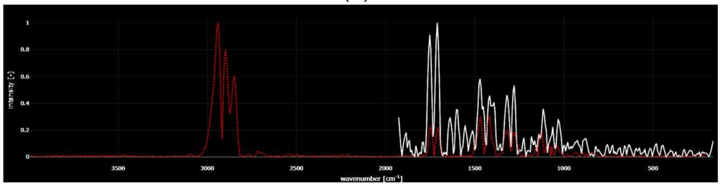

(c)

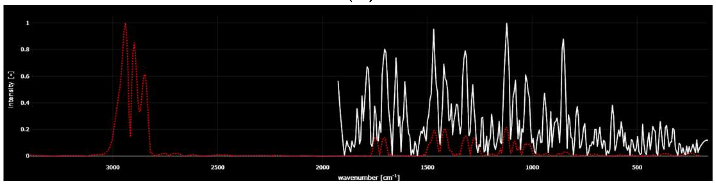

(d)

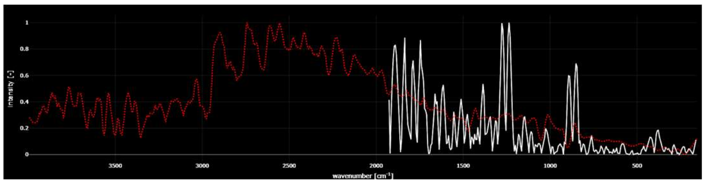

(e)

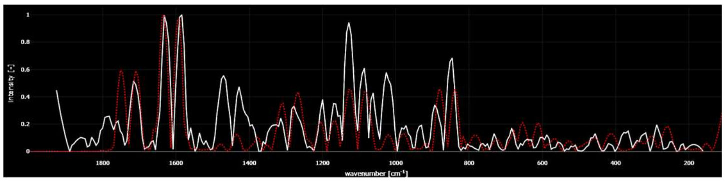

(f)

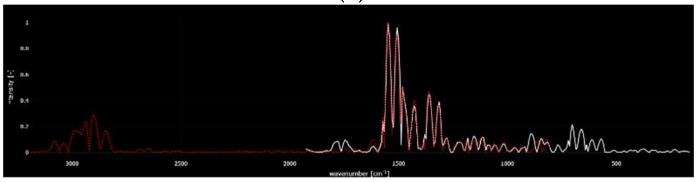

(g)

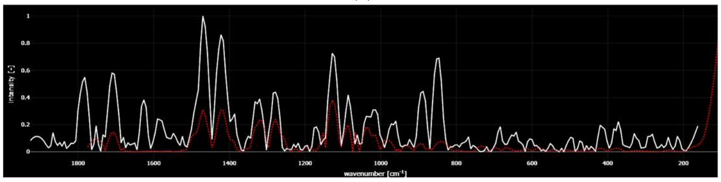

(h)

Supplemental Figure 1. Raman spectra analysis for : (a) PCL filament; (b) PLAPCL4060 filament; (c) PCL printed; (d) PLAPCL4060 printed; (e) PLA filament; (f) PLAPCL5050 filament; (g) PLA printed; and (h) PLAPCL5050 printed.
