# Supplementary material for: Pioneering Soundscapes: Investigating Commercial Fused Deposition Modelling Filament’s Potential for Ultrasound Technology in Bone Tissue Scaffolds
Source: Bioengineering (Basel). 2025 May 15;12(5):529. doi: 10.3390/bioengineering12050529 (PMC12108655; doi:10.3390/bioengineering12050529)
Supplement: Supplementary file 1 [file bioengineering-12-00529-s001.zip › This file provides the printing conditions that is set on the slicer program.pdf]

This file provides the printing conditions that is set on the slicer program (ideaMaker 5.6.0) and also the g-code file.

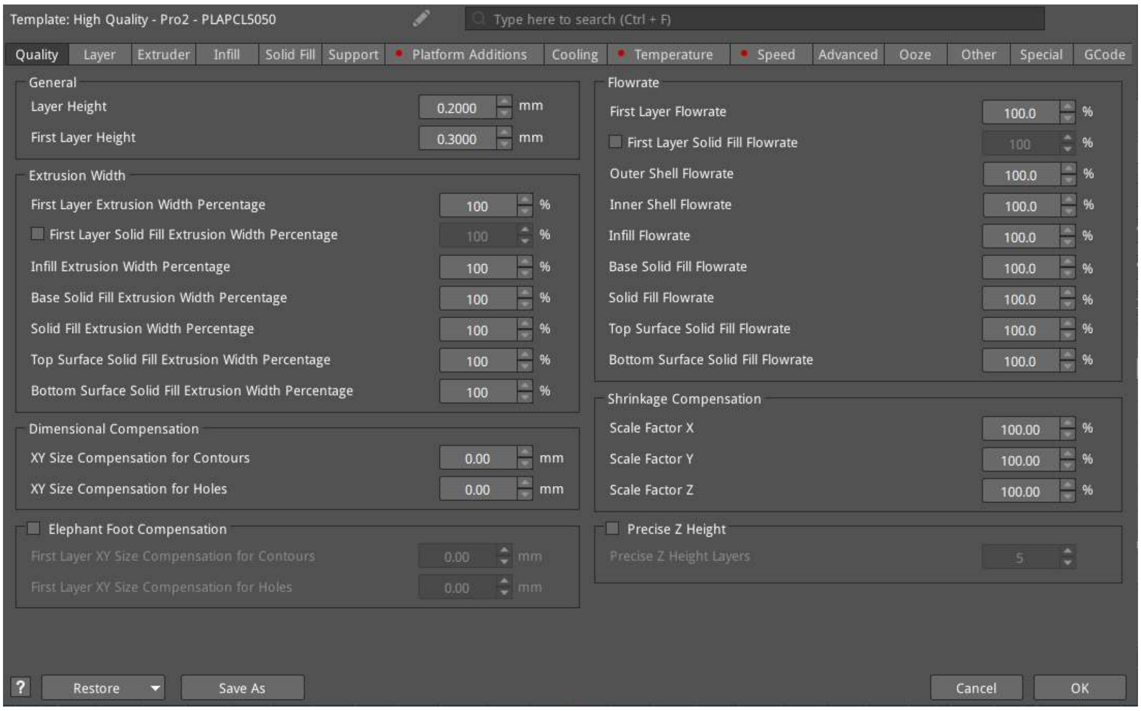

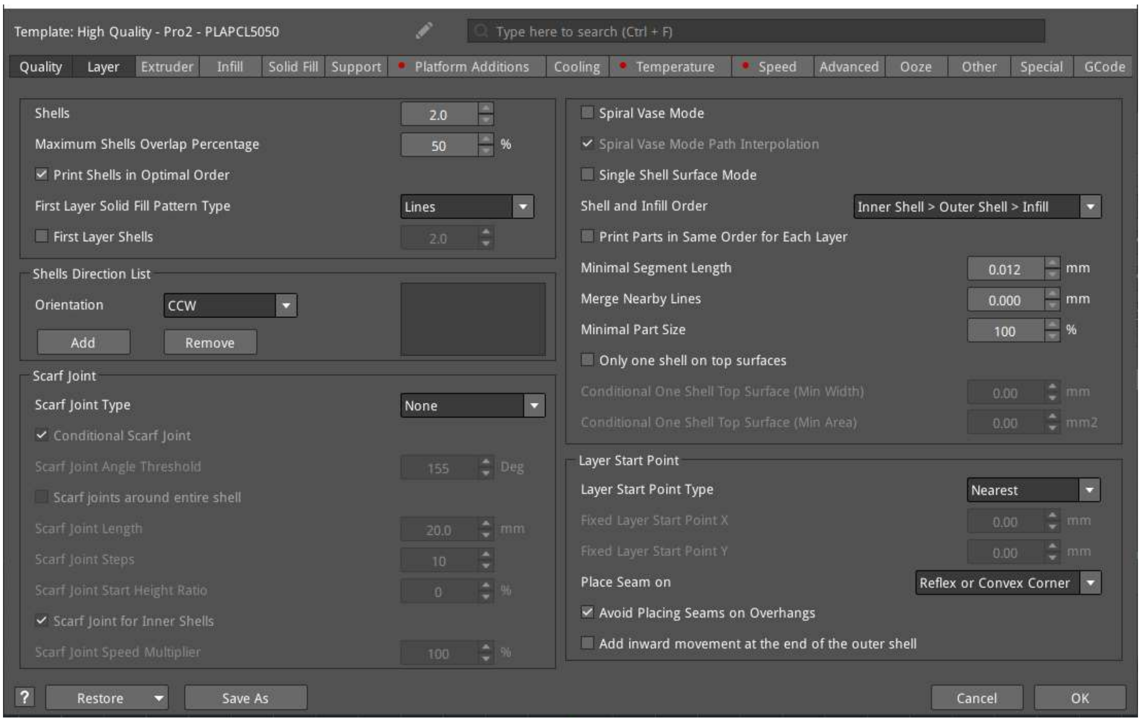

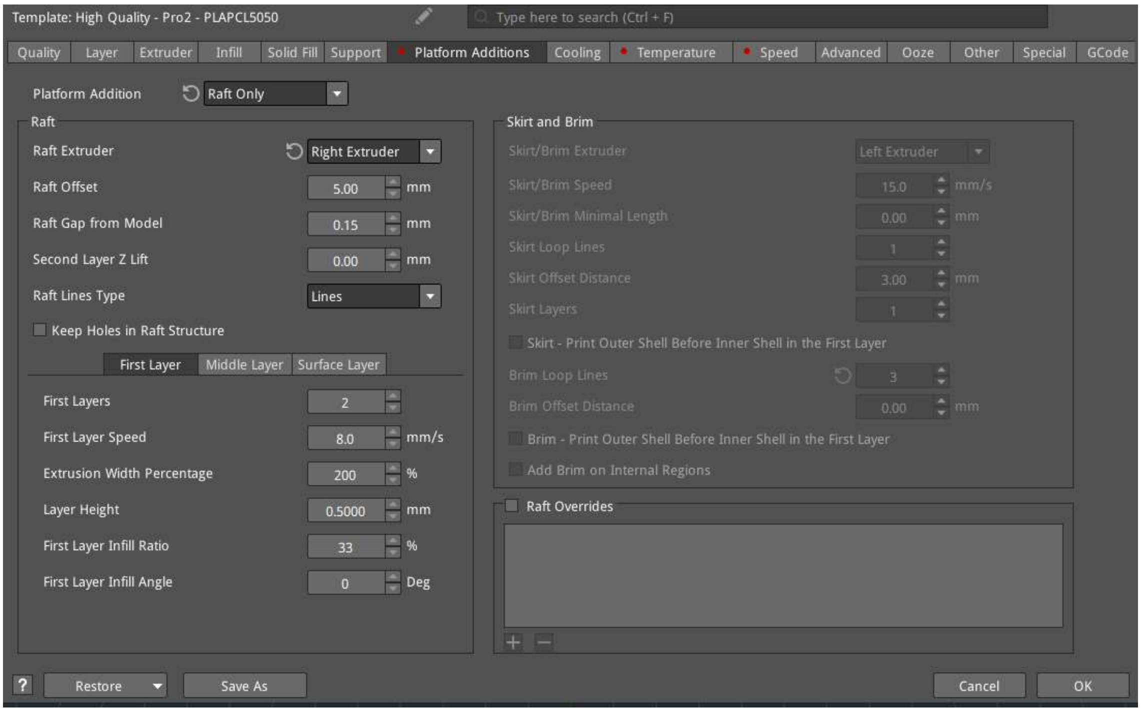
Template: High Quality - Pro2 - PLAPCL5050
Type here to search (Ctrl + F)
Quality
Layer
Extruder
Infill
Solid Fill
Support
Platform Additions
Cooling
Temperature
Speed
Advanced
Ooze
Other
Special
GCode
Platform Addition
Raft Only
Raft
Raft Extruder
Right Extruder
Raft Offset
5.00
mm
Raft Gap from Model
0.15
mm
Second Layer Z Lift
0.00
mm
Raft Lines Type
Lines
Keep Holes in Raft Structure
First Layer
Middle Layer
Surface Layer
First Layers
2
First Layer Speed
8.0
mm/s
Extrusion Width Percentage
200
%
Layer Height
0.5000
mm
First Layer Infill Ratio
33
%
First Layer Infill Angle
0
Deg
Skirt and Brim
Skirt/Brim Extruder
Left Extruder
Skirt/Brim Speed
15.0
mm/s
Skirt/Brim Minimal Length
0.00
mm
Skirt Loop Lines
1
Skirt Offset Distance
3.00
mm
Skirt Layers
1
Skirt - Print Outer Shell Before Inner Shell in the First Layer
Brim Loop Lines
3
Brim Offset Distance
0.00
mm
Brim - Print Outer Shell Before Inner Shell in the First Layer
Add Brim on Internal Regions
Raft Overrides
?
Restore
Save As
Cancel
OK

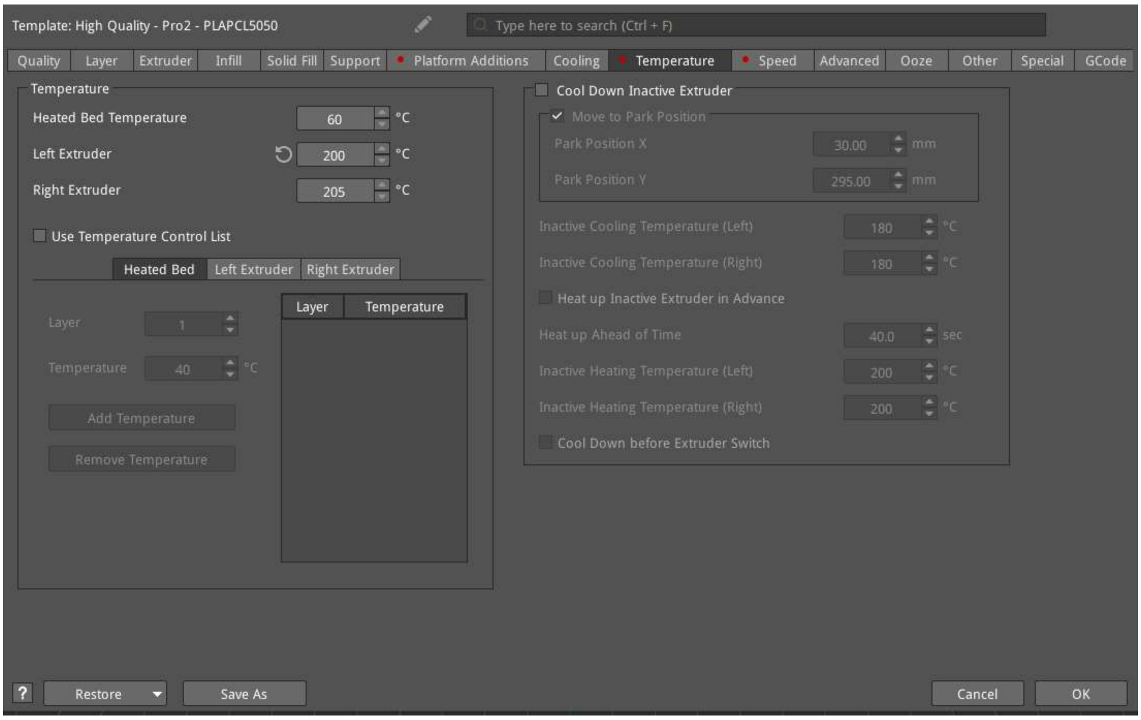
Template: High Quality - Pro2 - PLAPCL5050
Type here to search (Ctrl + F)
Quality
Layer
Extruder
Infill
Solid Fill
Support
Platform Additions
Cooling
Temperature
Speed
Advanced
Ooze
Other
Special
GCode
Temperature
Heated Bed Temperature
60
°C
Left Extruder
200
°C
Right Extruder
205
°C
Use Temperature Control List
Heated Bed
Left Extruder
Right Extruder
Layer
1
Temperature
40
°C
Add Temperature
Remove Temperature
Layer
Temperature
Cool Down Inactive Extruder
Move to Park Position
Park Position X
30.00
mm
Park Position Y
295.00
mm
Inactive Cooling Temperature (Left)
180
°C
Inactive Cooling Temperature (Right)
180
°C
Heat up Inactive Extruder in Advance
Heat up Ahead of Time
40.0
sec
Inactive Heating Temperature (Left)
200
°C
Inactive Heating Temperature (Right)
200
°C
Cool Down before Extruder Switch
?
Restore
Save As
Cancel
OK

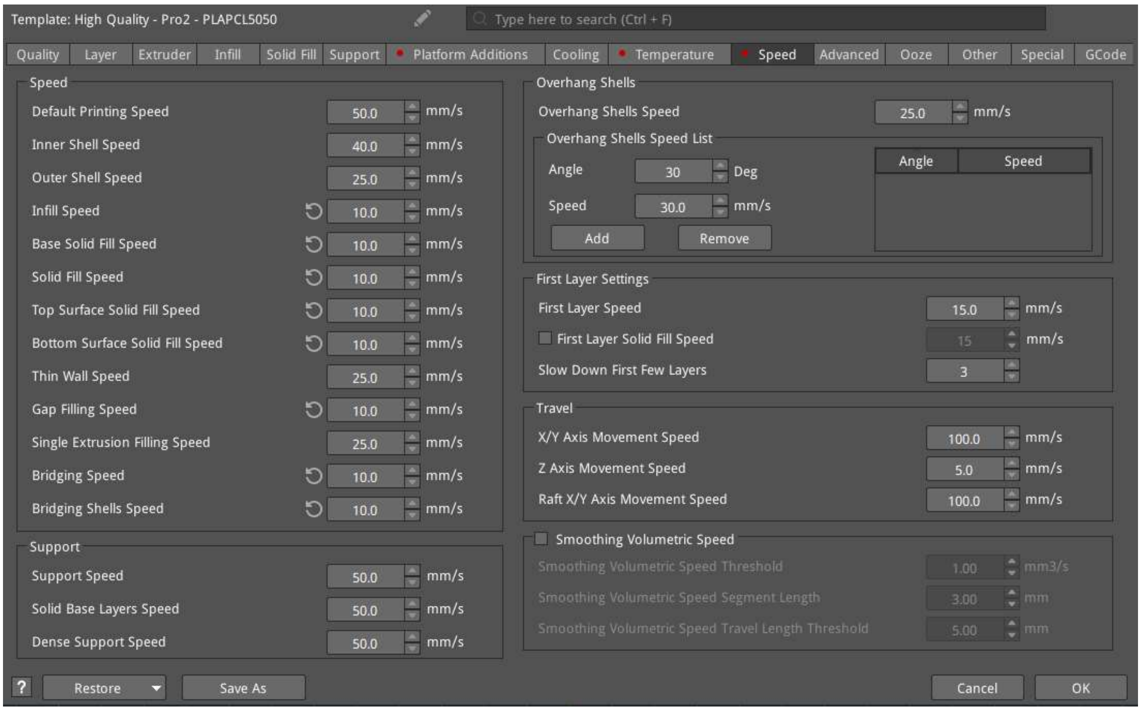
Template: High Quality - Pro2 - PLAPCL5050
Type here to search (Ctrl + F)
Quality
Layer
Extruder
Infill
Solid Fill
Support
Platform Additions
Cooling
Temperature
Speed
Advanced
Ooze
Other
Special
GCode
Speed
Default Printing Speed 50.0 mm/s
Inner Shell Speed 40.0 mm/s
Outer Shell Speed 25.0 mm/s
Infill Speed 10.0 mm/s
Base Solid Fill Speed 10.0 mm/s
Solid Fill Speed 10.0 mm/s
Top Surface Solid Fill Speed 10.0 mm/s
Bottom Surface Solid Fill Speed 10.0 mm/s
Thin Wall Speed 25.0 mm/s
Gap Filling Speed 10.0 mm/s
Single Extrusion Filling Speed 25.0 mm/s
Bridging Speed 10.0 mm/s
Bridging Shells Speed 10.0 mm/s
Support
Support Speed 50.0 mm/s
Solid Base Layers Speed 50.0 mm/s
Dense Support Speed 50.0 mm/s
Overhang Shells
Overhang Shells Speed 25.0 mm/s
Overhang Shells Speed List
Angle 30 Deg
Speed 30.0 mm/s
Add
Remove
Angle
Speed
First Layer Settings
First Layer Speed 15.0 mm/s
First Layer Solid Fill Speed 15 mm/s
Slow Down First Few Layers 3
Travel
X/Y Axis Movement Speed 100.0 mm/s
Z Axis Movement Speed 5.0 mm/s
Raft X/Y Axis Movement Speed 100.0 mm/s
Smoothing Volumetric Speed
Smoothing Volumetric Speed Threshold 1.00 mm3/s
Smoothing Volumetric Speed Segment Length 3.00 mm
Smoothing Volumetric Speed Travel Length Threshold 5.00 mm
?
Restore
Save As
Cancel
OK
