# Supplementary figures and images for: Pioneering Soundscapes: Investigating Commercial Fused Deposition Modelling Filament’s Potential for Ultrasound Technology in Bone Tissue Scaffolds
Source: Bioengineering (Basel). 2025 May 15;12(5):529. doi: 10.3390/bioengineering12050529 (PMC12108655; doi:10.3390/bioengineering12050529)

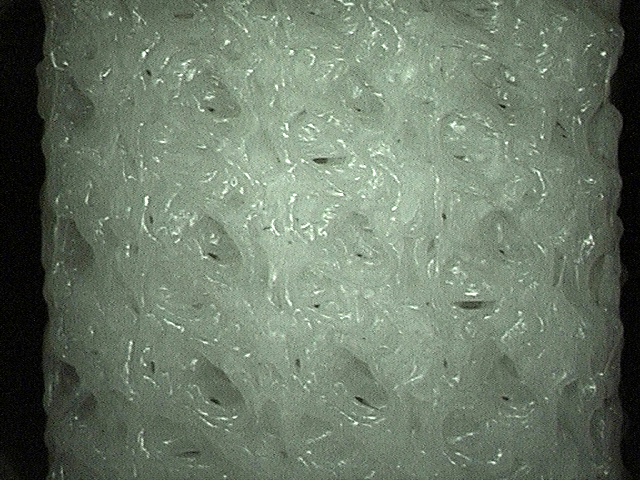

Supplement: Supplementary file 1 [file bioengineering-12-00529-s001.zip › Supplementary Figure A/Figure (a)/1_125.BMP]

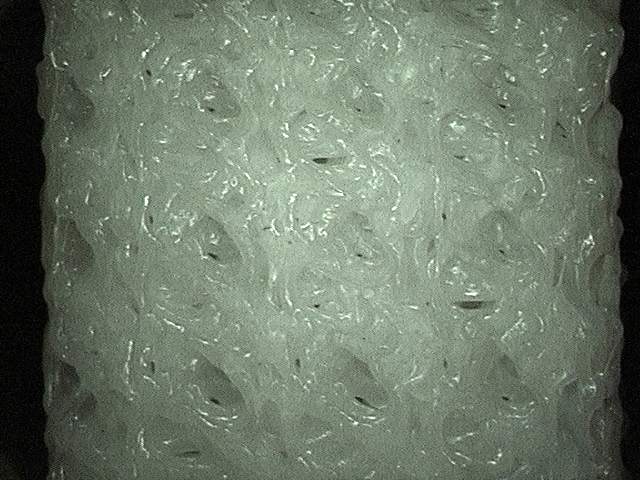

Supplement: Supplementary file 1 [file bioengineering-12-00529-s001.zip › Supplementary Figure A/Figure (a)/1_200.bmp]

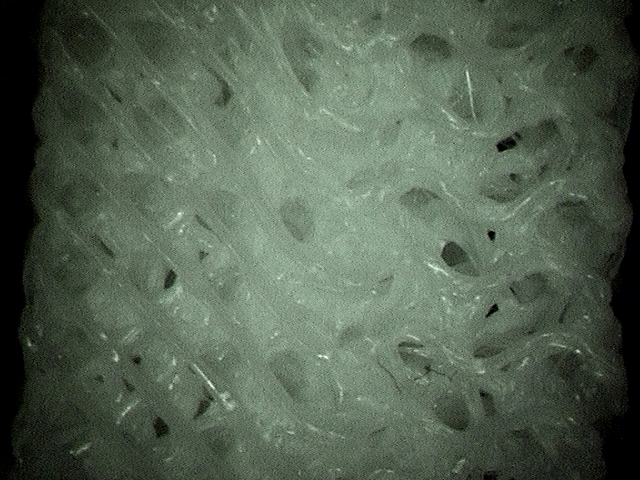

Supplement: Supplementary file 1 [file bioengineering-12-00529-s001.zip › Supplementary Figure A/Figure (a)/2_125.bmp]

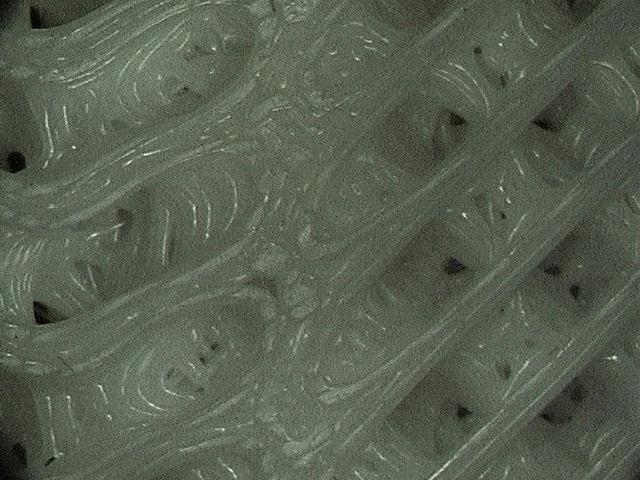

Supplement: Supplementary file 1 [file bioengineering-12-00529-s001.zip › Supplementary Figure A/Figure (a)/2_200.bmp]

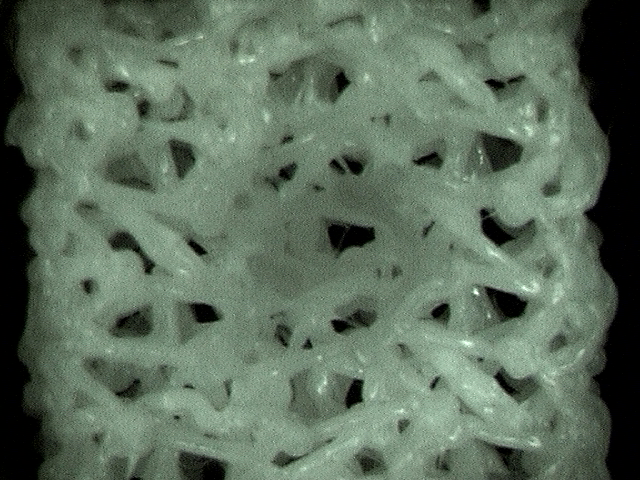

Supplement: Supplementary file 1 [file bioengineering-12-00529-s001.zip › Supplementary Figure A/Figure (a)/3_125.bmp]

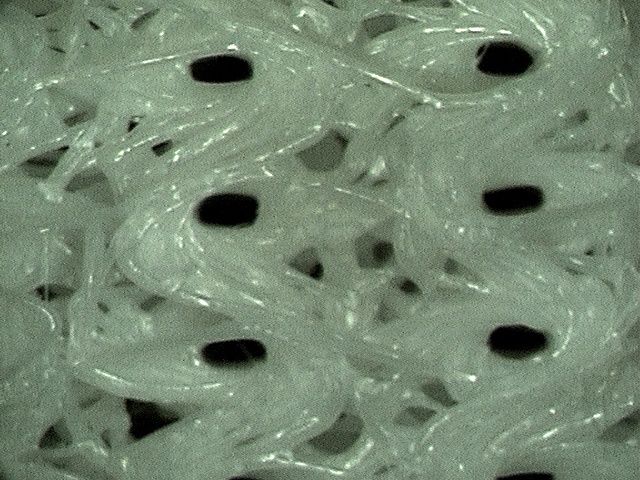

Supplement: Supplementary file 1 [file bioengineering-12-00529-s001.zip › Supplementary Figure A/Figure (a)/3_200.bmp]

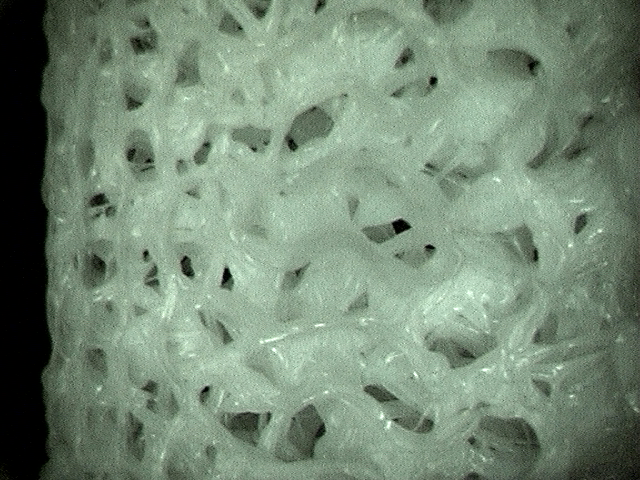

Supplement: Supplementary file 1 [file bioengineering-12-00529-s001.zip › Supplementary Figure A/Figure (a)/4_125.bmp]

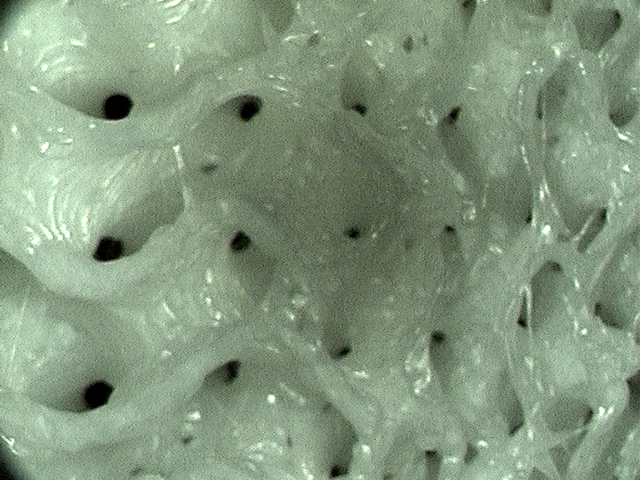

Supplement: Supplementary file 1 [file bioengineering-12-00529-s001.zip › Supplementary Figure A/Figure (a)/4_200.bmp]

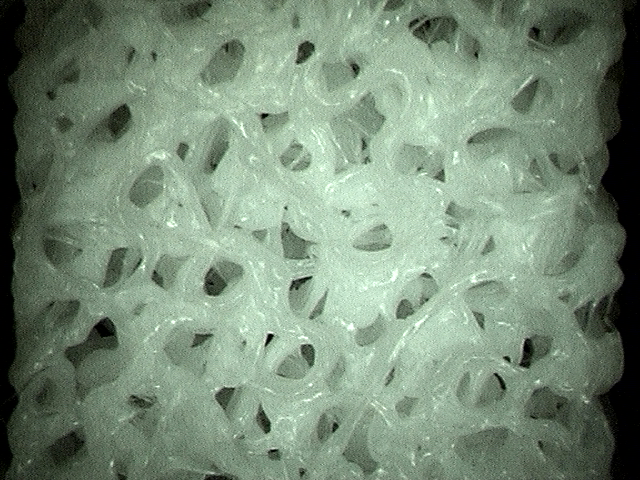

Supplement: Supplementary file 1 [file bioengineering-12-00529-s001.zip › Supplementary Figure A/Figure (a)/5_125.bmp]

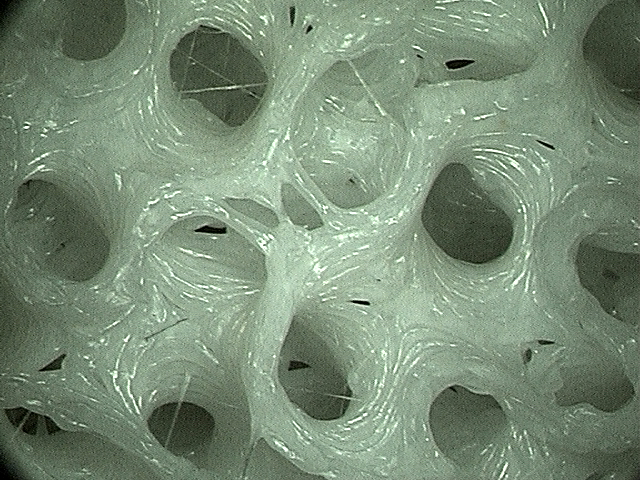

Supplement: Supplementary file 1 [file bioengineering-12-00529-s001.zip › Supplementary Figure A/Figure (a)/5_200.bmp]

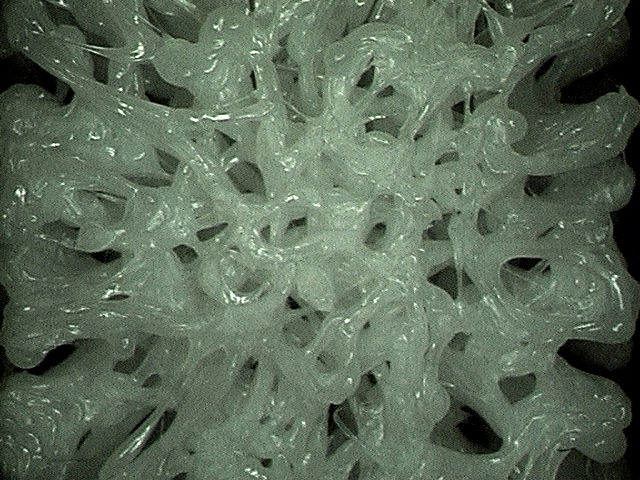

Supplement: Supplementary file 1 [file bioengineering-12-00529-s001.zip › Supplementary Figure A/Figure (a)/6_125.bmp]

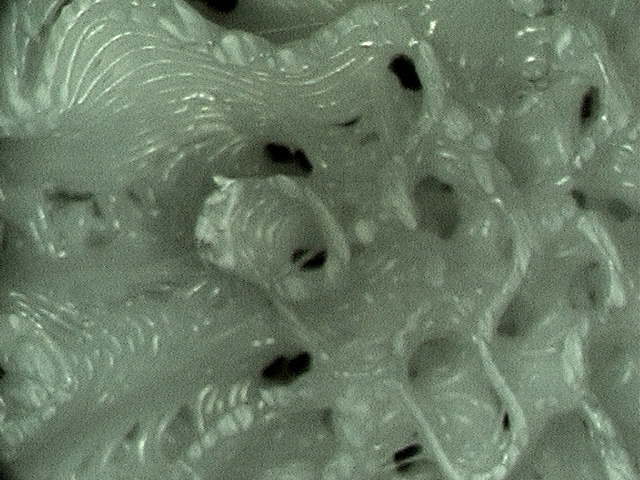

Supplement: Supplementary file 1 [file bioengineering-12-00529-s001.zip › Supplementary Figure A/Figure (a)/6_200.bmp]

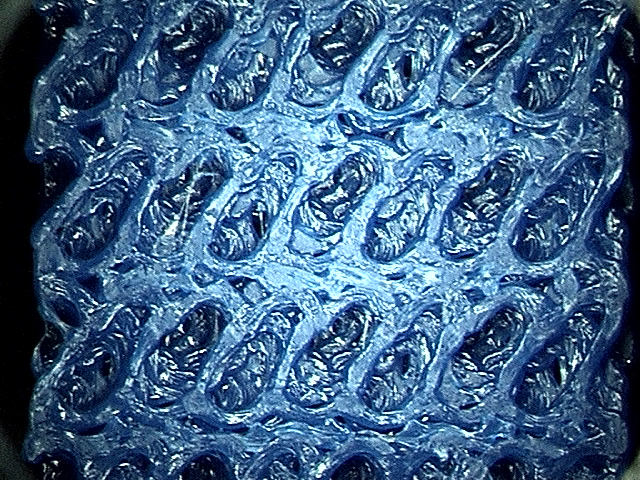

Supplement: Supplementary file 1 [file bioengineering-12-00529-s001.zip › Supplementary Figure A/Figure (b)/1_125.bmp]

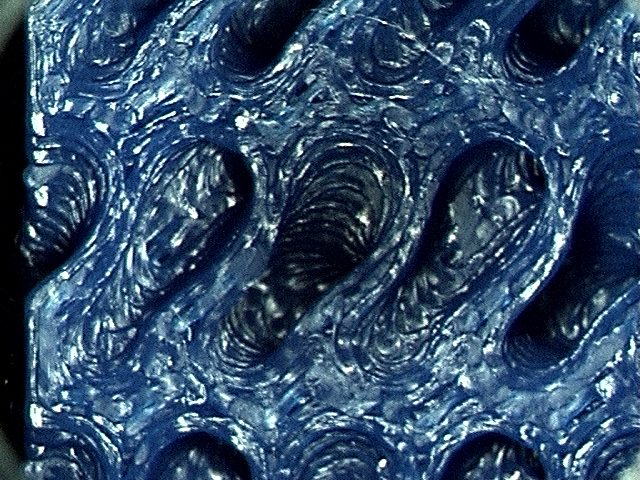

Supplement: Supplementary file 1 [file bioengineering-12-00529-s001.zip › Supplementary Figure A/Figure (b)/1_200.bmp]

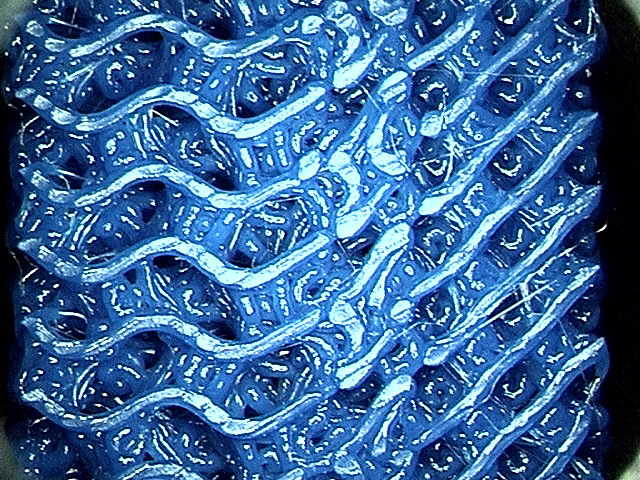

Supplement: Supplementary file 1 [file bioengineering-12-00529-s001.zip › Supplementary Figure A/Figure (b)/2_125.bmp]

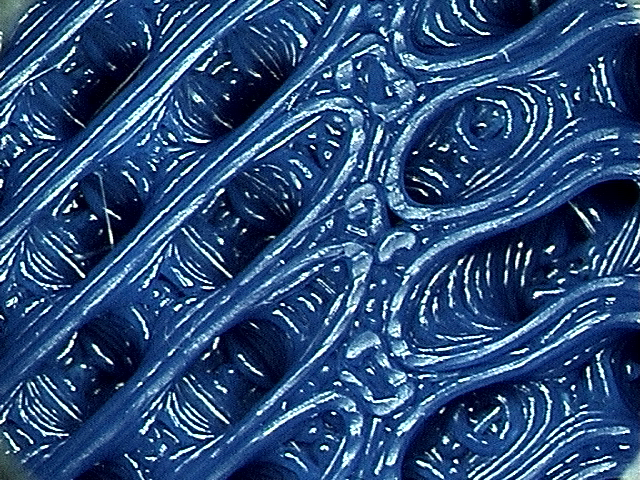

Supplement: Supplementary file 1 [file bioengineering-12-00529-s001.zip › Supplementary Figure A/Figure (b)/2_200.bmp]

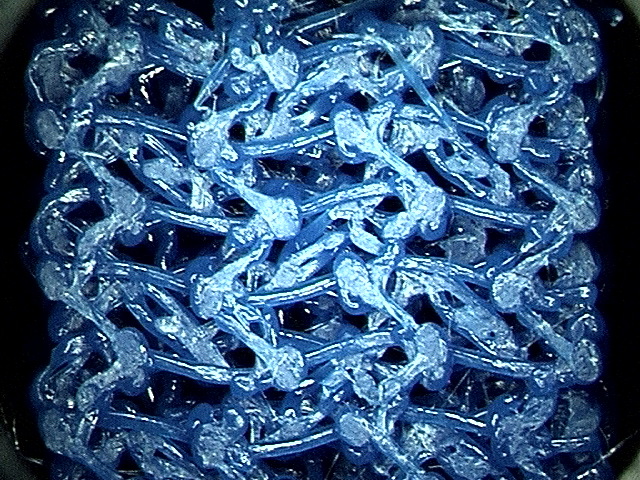

Supplement: Supplementary file 1 [file bioengineering-12-00529-s001.zip › Supplementary Figure A/Figure (b)/3_125.bmp]

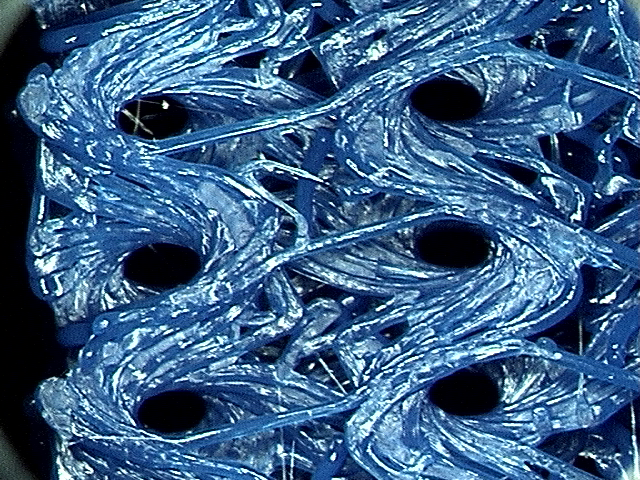

Supplement: Supplementary file 1 [file bioengineering-12-00529-s001.zip › Supplementary Figure A/Figure (b)/3_200.bmp]

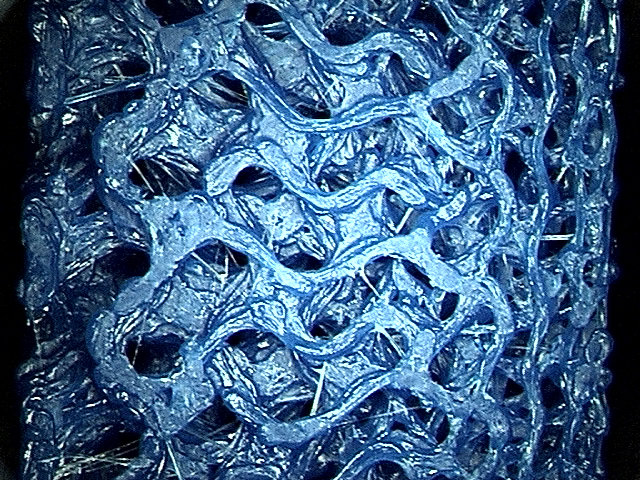

Supplement: Supplementary file 1 [file bioengineering-12-00529-s001.zip › Supplementary Figure A/Figure (b)/4_125.bmp]

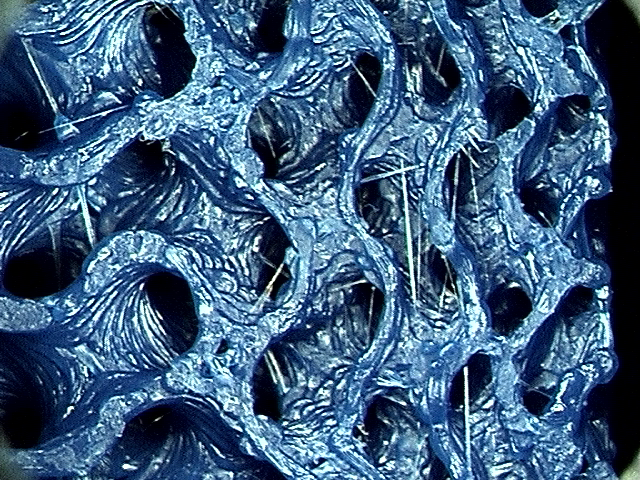

Supplement: Supplementary file 1 [file bioengineering-12-00529-s001.zip › Supplementary Figure A/Figure (b)/4_200.bmp]

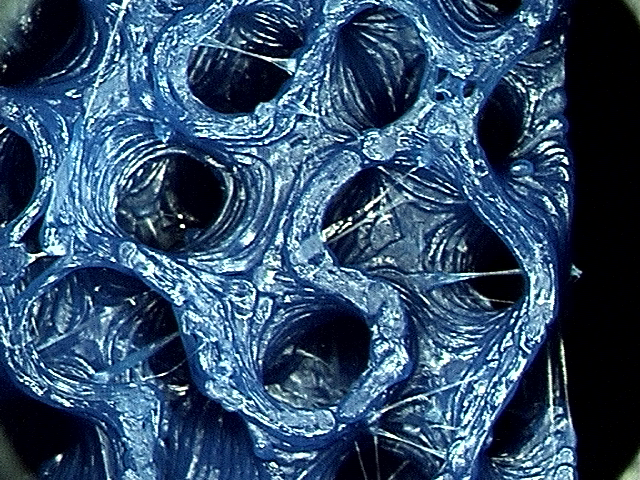

Supplement: Supplementary file 1 [file bioengineering-12-00529-s001.zip › Supplementary Figure A/Figure (b)/5_200.bmp]

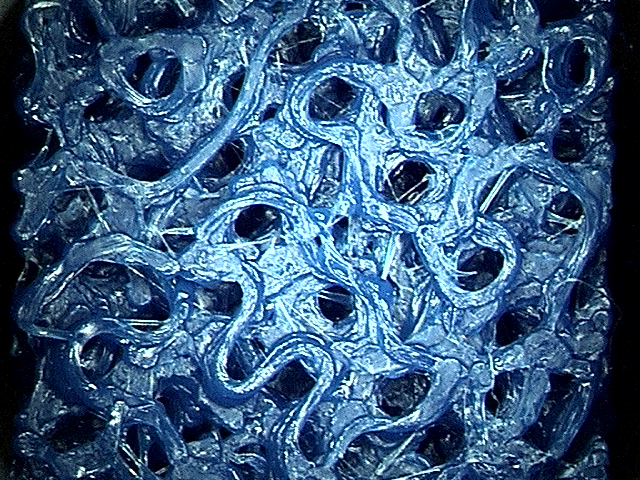

Supplement: Supplementary file 1 [file bioengineering-12-00529-s001.zip › Supplementary Figure A/Figure (b)/5_2125.bmp]

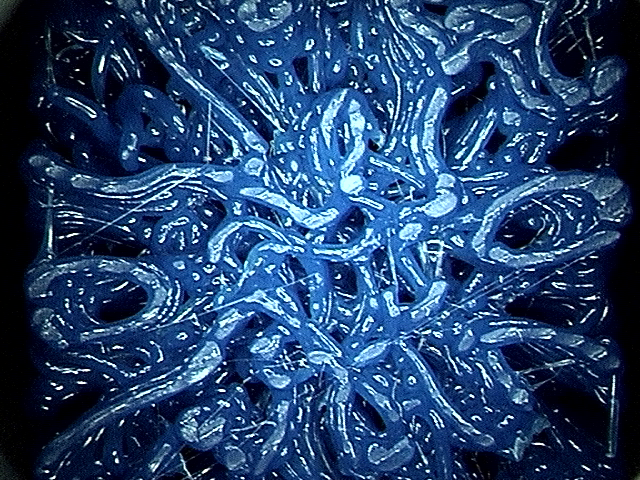

Supplement: Supplementary file 1 [file bioengineering-12-00529-s001.zip › Supplementary Figure A/Figure (b)/6_125.bmp]

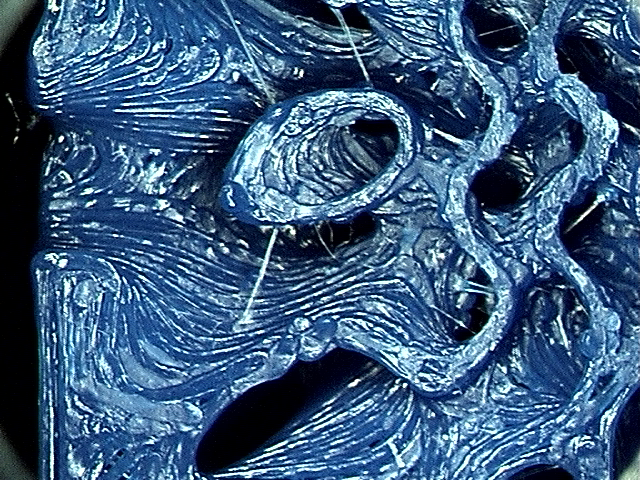

Supplement: Supplementary file 1 [file bioengineering-12-00529-s001.zip › Supplementary Figure A/Figure (b)/6_200.bmp]

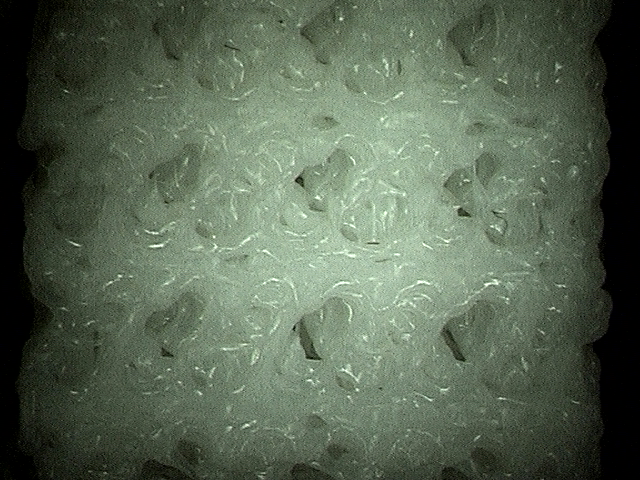

Supplement: Supplementary file 1 [file bioengineering-12-00529-s001.zip › Supplementary Figure A/Figure (c)/4060/1_125.bmp]

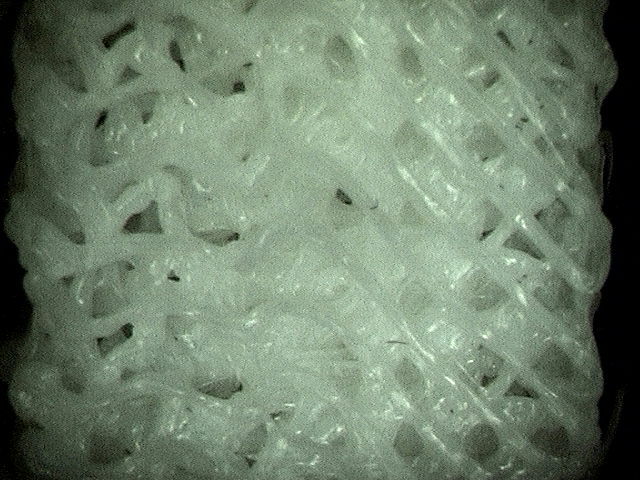

Supplement: Supplementary file 1 [file bioengineering-12-00529-s001.zip › Supplementary Figure A/Figure (c)/4060/2_125.bmp]

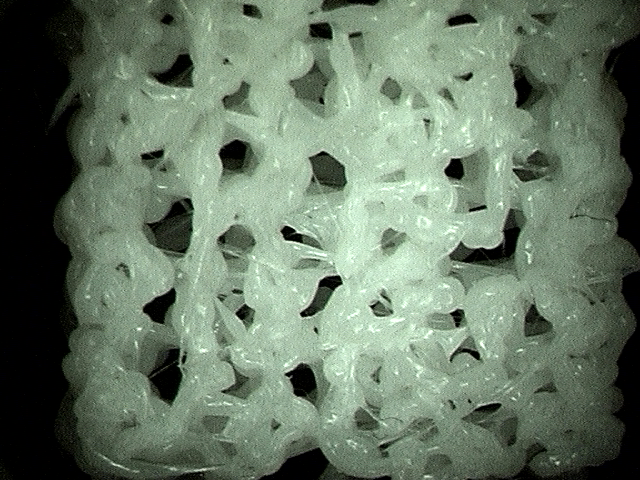

Supplement: Supplementary file 1 [file bioengineering-12-00529-s001.zip › Supplementary Figure A/Figure (c)/4060/3_125.bmp]

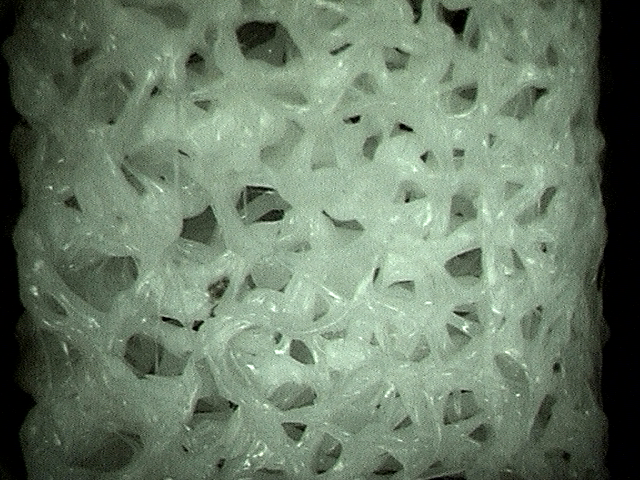

Supplement: Supplementary file 1 [file bioengineering-12-00529-s001.zip › Supplementary Figure A/Figure (c)/4060/4_125.bmp]

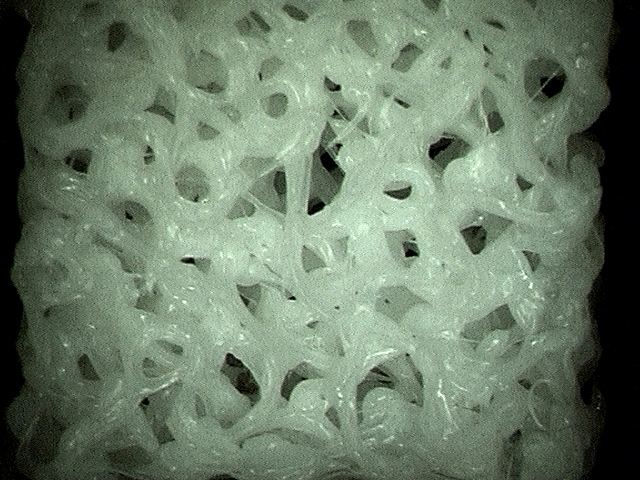

Supplement: Supplementary file 1 [file bioengineering-12-00529-s001.zip › Supplementary Figure A/Figure (c)/4060/5_125.bmp]

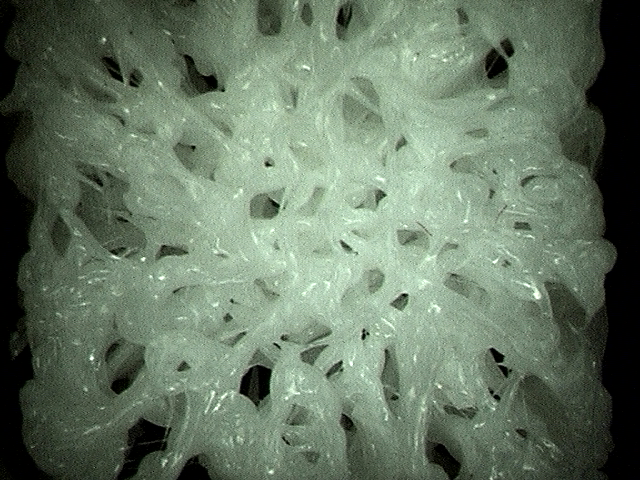

Supplement: Supplementary file 1 [file bioengineering-12-00529-s001.zip › Supplementary Figure A/Figure (c)/4060/6_125.bmp]

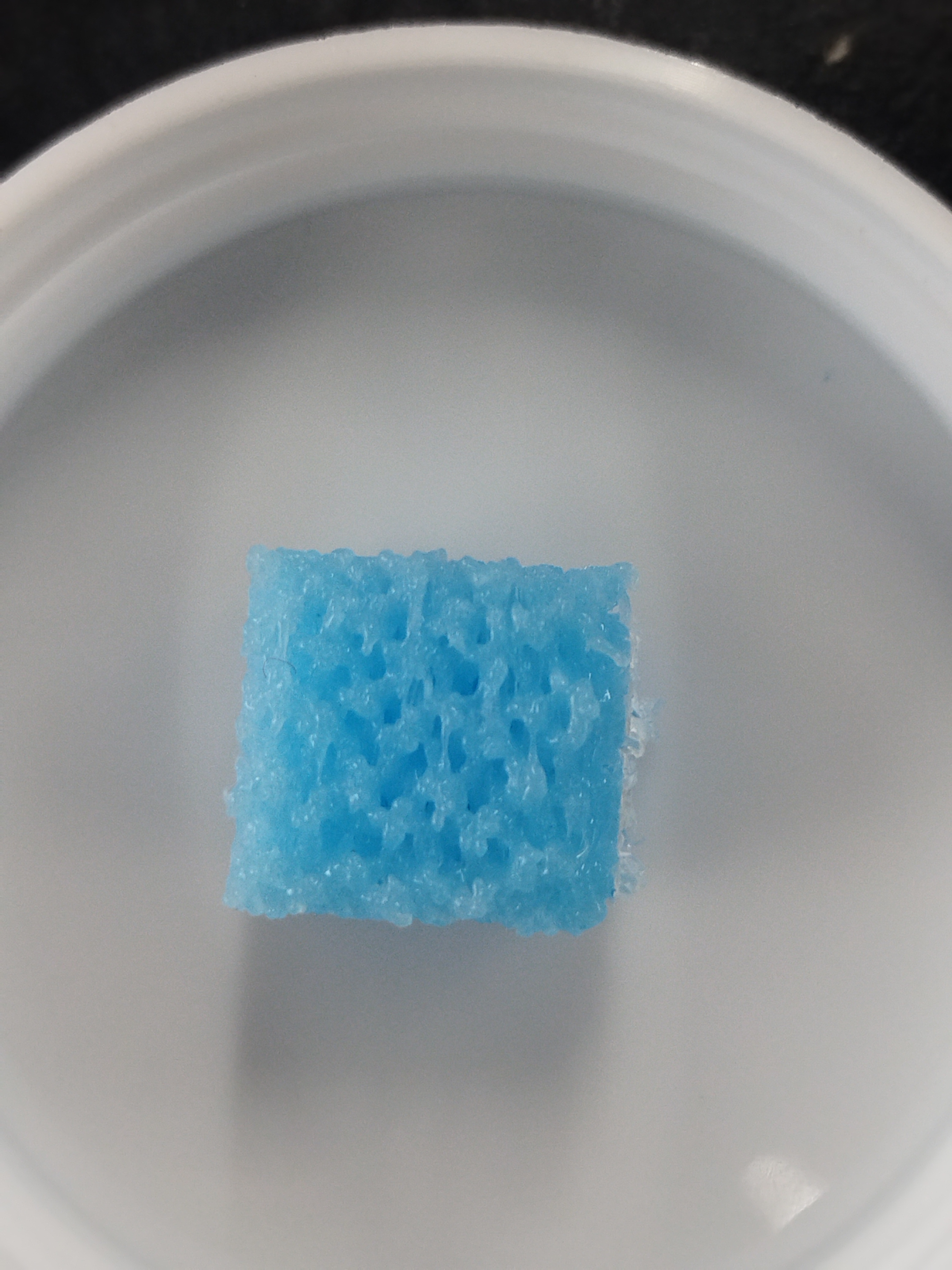

Supplement: Supplementary file 1 [file bioengineering-12-00529-s001.zip › Supplementary Figure B.jpg]
